# Examining the Protein Interactome and Subcellular Localization of RNase J2 Complexes in *Streptococcus mutans*

**DOI:** 10.3389/fmicb.2019.02150

**Published:** 2019-09-18

**Authors:** Rong Mu, Pushkar Shinde, Zhengzhong Zou, Jens Kreth, Justin Merritt

**Affiliations:** ^1^Department of Restorative Dentistry, School of Dentistry, Oregon Health and Science University, Portland, OR, United States; ^2^Emory College of Arts and Sciences, Atlanta, GA, United States; ^3^Department of Molecular Microbiology and Immunology, School of Medicine, Oregon Health and Science University, Portland, OR, United States

**Keywords:** ribonuclease J2, ribonuclease J1, RNA degradosome, coimmunoprecipitation, Gram-positive, *Streptococcus mutans*

## Abstract

Regulated RNA turnover is vital for the control of gene expression in all cellular life. In *Escherichia coli*, this process is largely controlled by a stable degradosome complex containing RNase E and a variety of additional enzymes. In the Firmicutes phylum, species lack RNase E and often encode the paralogous enzymes RNase J1 and RNase J2. Unlike RNase J1, surprisingly little is known about the regulatory function and protein interactions of RNase J2, despite being a central pleiotropic regulator for the streptococci and other closely related organisms. Using crosslink coimmunoprecipitation in *Streptococcus mutans*, we have identified the major proteins found within RNase J2 protein complexes located in the cytoplasm and at the cell membrane. In both subcellular fractions, RNase J2 exhibited the most robust interactions with RNase J1, while additional transient and/or weaker “degradosome-like” interactions were also detected. In addition, RNase J2 exhibits multiple novel interactions that have not been previously reported for any RNase J proteins, some of which were highly biased for either the cytoplasmic or membrane fractions. We also determined that the RNase J2 C-terminal domain (CTD) encodes a structure that is likely conserved among RNase J enzymes and may have an analogous function to the C-terminal portion of RNase E. While we did observe a number of parallels between the RNase J2 interactome and the *E. coli* degradosome paradigm, our results suggest that *S. mutans* degradosomes are either unlikely to exist or are quite distinct from those of *E. coli*.

## Introduction

RNase activity is an essential component of bulk RNA turnover, RNA processing, and post-transcriptional gene regulation. In *Escherichia coli*, most of this activity is dependent upon the essential endoribonuclease RNase E, which is a key component of the multi-protein complex referred to as the RNA degradosome. RNase E contains a highly conserved catalytic region and unstructured regions that serve as a scaffold for degradosome assembly as well as to target the degradosome to the cell membrane (Liou et al., 2001; Khemici et al., 2008). The composition of the *E. coli* degradosome has been well characterized and consists of a mixture of enzymes directly involved in RNA metabolism, such as the DEAD-box RNA helicase (RhlB) (Miczak et al., 1996; Py et al., 1996), polynucleotide phosphorylase (PNPase) (Miczak et al., 1996; Liou et al., 2001), polyphosphate kinase (PPK) (Blum et al., 1997), and RNase II (Lu and Taghbalout, 2014). In addition, the degradosome contains other proteins with unknown or possibly no direct roles in RNA metabolism, such as the glycolytic enzyme enolase (Eno) (Miczak et al., 1996; Chandran and Luisi, 2006) and the heat shock protein DnaK (Miczak et al., 1996). Many bacteria and archaea, including most Gram-positive bacteria, do not encode RNase E, and instead encode one or more RNase J paralogs (Bandyra et al., 2013; Clouet-d’Orval et al., 2015). For the Firmicutes, two RNase J paralogs referred to as RNase J1 and RNase J2 are typically encoded. In *Bacillus subtilis*, both RNases J1 and J2 possess 5′-3′ exoribonucleolytic activity, but RNase J1 activity is substantially stronger compared to RNase J2 (Mathy et al., 2010). The *B. subtilis* RNase J1 also has endoribonuclease activity *in vitro*, but it is unclear whether this activity is physiologically relevant (Even et al., 2005; Mathy et al., 2010; Newman et al., 2011). In addition, many RNase J-encoding species also encode the membrane localized endoribonuclease RNase Y. Similar to RNase J1 mutants, a deletion of RNase Y in *B. subtilis* results in pleiotropic effects, including a >2-fold increase in bulk mRNA stability (Shahbabian et al., 2009). Degradosome-like protein complexes have been detected with RNase Y and include interactions with RNases J1/J2, PNPase, DEAD-box RNA helicase (CshA), and the glycolytic enzymes enolase (Eno) and phosphofructokinase (PfkA) (Lehnik-Habrink et al., 2010, 2011). Similar degradosome-like components were detected in *Staphylococcus aureus* as well. Using bacterial two-hybrid analyses, binary interactions were observed between RNases J1/J2, RNase J1/PNPase, RNase Y/Enolase, and RNase Y/RNA helicase (Roux et al., 2011). Currently, it is unclear whether these interactions represent the basal state of a stable degradosome-like complex, a degradosome-like complex with variable transient interactions, or if these interactions are indicative of multiple distinct protein complexes (Redder, 2018). The evidence suggests that at a minimum, RNases J1 and J2 form stable heterotetramers (Mathy et al., 2010). Interestingly, RNase J1/J2 complexes also exhibit distinct enzymatic activities compared to the individual enzymes (Mathy et al., 2010). In contrast to RNases J1 and Y, surprisingly little is known about the protein interactome and regulatory role of RNase J2. In *B. subtilis*, RNase J2 is only known to associate in degradosome-like complexes via interactions with RNases J1 and Y (Mathy et al., 2010; Lehnik-Habrink et al., 2011; Figaro et al., 2013), whereas in *Staphylococcus epidermidis* an additional interaction with PNPase was proposed based upon *in vitro* binding studies (Raj et al., 2018). However, an RNase J2-PNPase interaction was not detected in the aforementioned *S. aureus* bacterial two-hybrid study (Roux et al., 2011). Phenotypically, RNase J2 appears to be largely dispensable in *B. subtilis*, whereas RNase J1 mutants exhibit pleiotropic effects, including significantly slowed growth (Even et al., 2005; Figaro et al., 2013). In contrast, RNase J2 appears to be of equal or greater importance to RNase J1 in *S. aureus*, *Streptococcus pyogenes*, and *Streptococcus mutans*. In *S. aureus*, a deletion of either RNase J1 or J2 yields similar growth defects and altered RNA cleavage patterns suggesting they both target similar transcripts (Linder et al., 2014). Likewise, in *S. pyogenes*, both RNases J1 and J2 are essential for cell growth and have largely overlapping target specificities (Bugrysheva and Scott, 2010). These results parallel our previous observations in *S. mutans*. While RNase J1 and J2 mutants both exhibit poor growth and pleiotropic effects, key phenotypes such as growth rate, morphology, biofilm formation, and environmental stress tolerance were more severe in the RNase J2 mutant compared to either an RNase J1 mutant or an RNase J1/J2 double mutant (Chen et al., 2015). The putative catalytic center of RNase J2 in *S. mutans* and other closely related species also share a greater similarity to the RNase J1 consensus (HxHxDH) compared to the *B. subtilis* RNase J2 (Figaro et al., 2013; Chen et al., 2015). Accordingly, the *S. mutans* RNase J2 exhibits potent exo- and endoribonuclease activity *in vitro* (Liu et al., 2015). Despite the highly distinct roles of RNase J2 in *B. subtilis* and *S. mutans*, RNase J2 modulates the activity of RNase J1 in both species (Mathy et al., 2010; Chen et al., 2015), which suggests that this is a highly conserved feature of the RNase J1/J2 interaction.

In addition to its composition, membrane localization is another defining feature of the *E. coli* degradosome. RNase E is a membrane associated protein that binds to the cell membrane via an amphipathic helix as well as multiple regions of net positive charge (Khemici et al., 2008; Murashko et al., 2012). In RNase J-encoding species, only RNase Y has been shown to be directly membrane associated (Lehnik-Habrink et al., 2011; Cascante-Estepa et al., 2016). While multiple RNA metabolizing enzymes interact with RNase Y in *B. subtilis* (Commichau et al., 2009), it is not yet clear whether these interactions mediate stable membrane localization comparable to the role of RNase E (Khemici et al., 2008; Gorna et al., 2012). Furthermore, unlike the *E. coli* degradosome, RNase J degradosome-like complexes do not exhibit a similarly strict bias for membrane localization (Cascante-Estepa et al., 2016). Given the limited knowledge about RNase J2 protein interactions and subcellular localization, we performed a variety of crosslink coimmunoprecipitation (co-IP) and fractionation studies in *S. mutans*. Our results identified a number of degradosome-like interactions as well as several novel interactions that have not been previously reported. However, most interactions appeared to be weak/transient, except for that of the RNase J1/J2 complex. Furthermore, both the N- and C-terminal domains of RNase J2 are able to localize to the cell membrane, while the CTD also serves as a site of multiple protein–protein interactions.

## Results

### RNase J2 Abundance and Subcellular Localization in *S. mutans*

**FIGURE 1:**
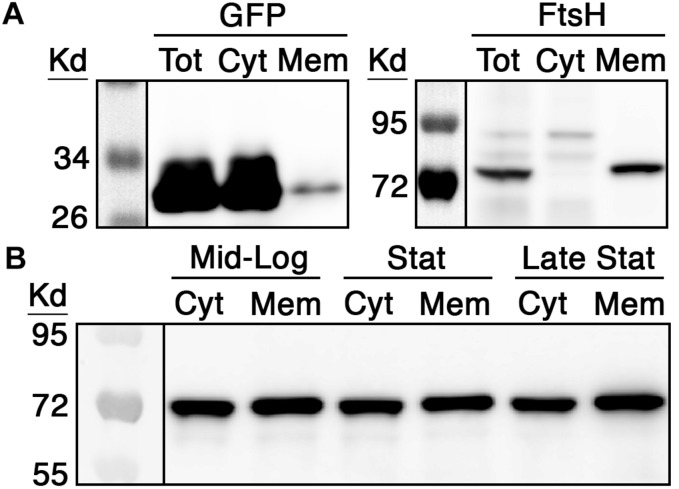
RNase J2 expression characteristics and subcellular localization. **(A)** Both green fluorescent protein (GFP) and the housekeeping protease FtsH were employed as controls to assess the efficacy of cytoplasmic and membrane protein fractionation via ultracentrifugation. To detect the localization of GFP, the wild-type strain UA159 was transformed with a multicopy vector encoding a 3x FLAG tagged GFP under the control of a highly expressed constitutive promoter. To detect FtsH localization, DNA encoding a FLAG tagged FtsH was inserted into the xylose-inducible expression vector pZX9 and then transformed into UA159. Cultures of both strains were grown to mid-log phase and then total protein (Tot) was fractionated to collect cytoplasmic (Cyt) and membrane (Mem) fractions. The resulting protein fractions were analyzed by western blot with anti-FLAG antibody. **(B)** The chromosomal copy of *S. mutans rnjB* (RNase J2) was modified to encode a C-terminal 3x FLAG epitope and then RNase J2 abundance was measured at mid-logarithmic phase (Mid-Log; OD_600_ 0.5), early stationary phase (Stat; OD_600_ 1.0), and late stationary phase (Late Stat; overnight growth). 40 μg of cytoplasmic (Cyt) and membrane (Mem) fraction extracts from each growth phase were analyzed by western blot with anti-FLAG antibody.

Stable membrane localization is both constitutive and essential for the proper functioning of the *E. coli* degradosome (Khemici et al., 2008; Strahl et al., 2015; Hadjeras et al., 2019), whereas the localization characteristics of RNase J enzymes are still unclear. Therefore, we were curious to first examine RNase J2 abundance and localization in *S. mutans* throughout its growth phase. To first validate our cellular fractionation protocol, we performed differential ultracentrifugation on protein lysates to separate cytoplasmic and membrane fractions to compare the localization of both green fluorescent protein (GFP) and FtsH. As expected, the vast majority of GFP protein was detected in the cytoplasmic fraction with only a faint signal present in the membrane fraction, whereas the housekeeping membrane protease FtsH was only detectable in the membrane fraction (Figure 1A). Next, we repeated this same fractionation protocol using a wild-type *S. mutans* strain expressing a chromosomally encoded RNase J2 containing a C-terminal 3x FLAG tag. We have previously demonstrated that RNase J2 is highly amenable to both N- and C-terminal fusions without triggering detectable changes in growth rate or other deleterious effects indicative of impaired function (Liu et al., 2015, 2017). Cells were collected at mid log phase (OD_600_ = 0.5), early stationary phase (OD_600_ = 1.0), and late stationary phase (overnight). As shown in Figure 1B, RNase J2 is stably and constitutively produced and remains similarly abundant in both the cytoplasmic and membrane fractions irrespective of growth phase. The strong signals present in the membrane fraction confirmed that a large portion of RNase J2 is indeed membrane localized. However, similarly strong signals were present in the cytoplasmic fraction during all growth phases, indicating that RNase J2 is not exclusively membrane associated, but is prominently distributed in both fractions (Figure 1B).

### Candidate RNase J2 Protein Interactions Identified by Crosslink Coimmunoprecipitation and Mass Spectrometry

To identify potential high and low affinity protein interactions with RNase J2, we first crosslinked mid-log phase cultures *in vivo* with paraformaldehyde, then purified RNase J2 protein complexes from cytoplasmic and membrane fractions using FLAG affinity resin, and lastly analyzed the immunopurified complexes via mass spectrometry. Specific enrichment was calculated by comparing the mass spectrometry spectral count values (total protein abundance) for individual proteins detected from both the C-terminal FLAG tagged RNase J2 strain vs. the parent wild-type *S. mutans* (i.e., no FLAG epitope). Among all of the detected proteins co-purified with RNase J2, RNase J1 yielded the most robust interaction. In fact, its spectral count and enrichment values were nearly identical to that of the immunoprecipitated (IP) bait protein RNase J2 in both the cytoplasmic and membrane fractions (Supplementary Table S3). This suggests both proteins primarily form highly stable heterodimers regardless of subcellular location. More than 500 additional proteins exhibited ≥2-fold enrichment in one or both fractions, albeit most had weak spectral count values and were therefore considered unreliable. Regardless, >100 candidate proteins did exhibit both reliable spectral count values and ≥2-fold enrichment. Of these, only L-lactate dehydrogenase (Ldh) yielded values close to that of RNase J1 (Supplementary Table S3).

### Validation of RNase J2 Mediated Protein Interactions *in vivo*

**FIGURE 2:**
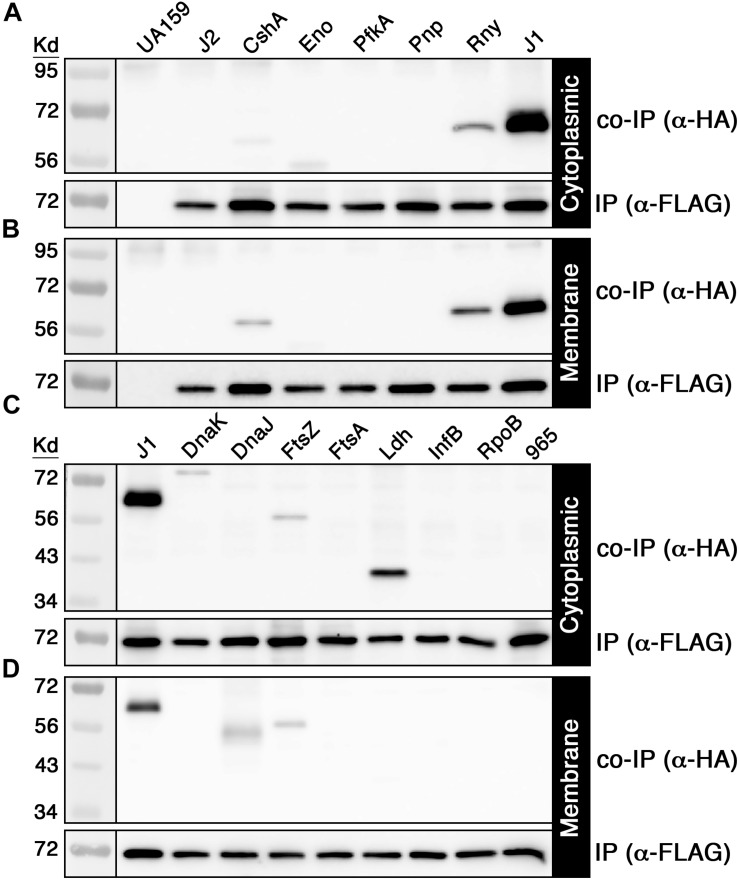
Coimmunoprecipitation of candidate RNase J2 protein interactions. 3x FLAG tagged RNase J2 was used as a bait to coimmunoprecipitate HA tagged proteins from both cytoplasmic and membrane protein fractions. All reactions were immunoprecipitated using anti-FLAG affinity resin. Samples in the top panels represent the coimmunoprecipitated samples probed with anti-HA antibody, while the bottom panels indicate the immunoprecipitated samples probed with anti-FLAG antibody. The unmodified wild-type (UA159) and the RNase J2-FLAG (J2) strains served as negative controls to assess reaction specificity. **(A)** Cytoplasmic fraction and **(B)** membrane fraction co-IP results with putative degradosome-like proteins. Strains from left to right: unmodified wild-type (UA159), RNase J2-FLAG (J2), RNase J2-FLAG + RNA helicase-HA (CshA), RNase J2-FLAG + Enolase-HA (Eno), RNase J2-FLAG + Phosphofructokinase-HA (PfkA), RNase J2-FLAG + PNPase-HA (Pnp), RNase J2-FLAG + RNase Y-HA (RnY), and RNase J2-FLAG + RNase J1-HA (J1). **(C)** Cytoplasmic fraction and **(D)** membrane fraction co-IP results with novel candidate RNase J2 protein interactions. Strains from left to right: RNase J2-FLAG + RNase J1-HA (J1), RNase J2-FLAG + DnaK-HA (DnaK), RNase J2-FLAG + DnaJ-HA (DnaJ), RNase J2-FLAG + FtsZ-HA (FtsZ), RNase J2-FLAG + Lactate dehydrogenase-HA (Ldh), RNase J2-FLAG + Translation initiation factor IF-2-HA (InfB), RNase J2-FLAG + RpoB-HA (RpoB), and RNase J2-FLAG + SMU_965-HA (965).

**FIGURE 3:**
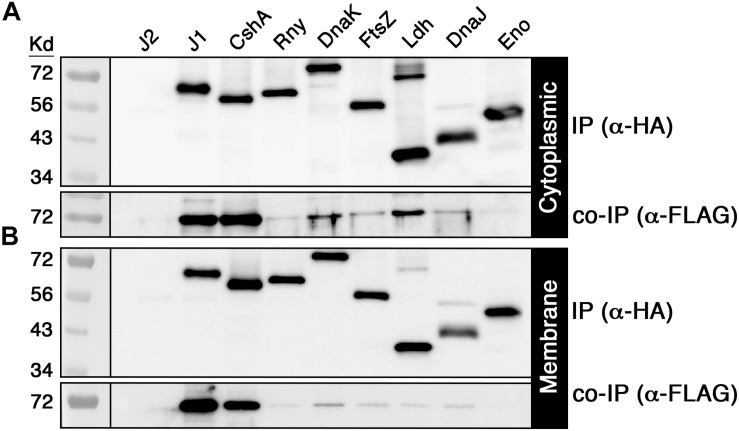
Validation of positive RNase J2 protein interactions. HA tagged proteins that were previously coimmunoprecipitated with RNase J2 were used as baits to coimmunoprecipitate RNase J2 from both cytoplasmic and membrane fractions. All reactions were immunoprecipitated using anti-HA affinity resin. Samples in the top panels represent the immunoprecipitated samples probed with anti-HA antibody, while the bottom panels indicate the coimmunoprecipitated samples probed with anti-FLAG antibody. The RNase J2-FLAG (J2) strain served as a negative control to assess reaction specificity. Both the **(A)** cytoplasmic fractions and **(B)** membrane fractions were analyzed. Samples from left to right: RNase J2-FLAG (J2), RNase J2-FLAG + RNase J1-HA (J1), RNase J2-FLAG + RNA Helicase-HA (CshA), RNase J2-FLAG + RNase Y-HA (Rny), RNase J2-FLAG + DnaK-HA (DnaK), RNase J2-FLAG + FtsZ-HA (FtsZ), RNase J2-FLAG + Lactate dehydrogenase-HA (Ldh), RNase J2-FLAG + DnaJ-HA (DnaJ), and RNase J2-FLAG + Enolase-HA (Eno).

Given the large number of potential RNase J2 protein interactions identified by mass spectrometry (Supplementary Table S3), we selected a subset of 14 candidate interacting proteins to determine whether the mass spectrometry results were independently verifiable. Each of these proteins was modified to express a C-terminal HA epitope tag and subsequently assayed via coimmunoprecipitation (co-IP) with the FLAG tagged RNase J2 serving as the bait protein. For the IP samples, the RNase J2-FLAG strain was used as a positive control, while the RNase J2-FLAG/RNase J1-HA strain served as a co-IP positive control. The unmodified wild-type strain UA159 served as a negative control for both the IP and co-IP samples. The candidate proteins selected from the Supplementary Table S3 dataset were assayed in two groups: the first consisted of proteins previously reported to exist in RNase J degradosome-like complexes, while the second group was selected to sample a diversity of spectral count/enrichment values and/or predicted protein functions. In agreement with the mass spectrometry results, RNase J1 and lactate dehydrogenase (Ldh) were among the strongest RNase J2 interactions detected via co-IP (Figures 2A–D). Of the reported degradosome-like proteins we examined, interactions were detectable with both RNase Y (RnY) and RNA helicase (CshA) along with a potential weak interaction with enolase (Eno) (Figures 2A,B). For the second group, the aforementioned Ldh interaction was detected in addition to weaker interactions with the heat shock proteins DnaK and DnaJ as well as the cell division GTPase FtsZ (Figures 2C,D). The Ldh interaction was strongly biased for the cytoplasmic fraction, whereas DnaK and DnaJ signals were specific to the cytoplasmic and membrane fractions, respectively. We found no evidence for complex formation between RNase J2 and phosphofructokinase (PfkA), PNPase, the cell division protein FtsA, translation initiation factor IF-2 (InfB), RNA polymerase beta subunit (RpoB), or the putative homoserine dehydrogenase SMU_965. As a final confirmation, we performed a reciprocal HA IP using each of the positive interactors identified in Figure 2 as bait proteins to co-purify RNase J2. Overall, the results largely mirrored those of the FLAG IP, except that the HA IP resulted in a stronger interaction with RNA helicase (CshA) and a weaker interaction with RNase Y (Figures 3A,B). Taken together, the co-IP results largely support the findings of the original mass spectrometry proteomic screen, as both previously reported and novel RNase J2 interactions identified in the mass spectrometry dataset were detectable via specific co-IP assays. A portion of the tested interactions identified in Supplementary Table S3 were not reproducible in our co-IP assays, which suggests that some false positives might exist in the mass spectrometry data, particularly for proteins exhibiting weak spectral count and/or enrichment values. Based upon the mass spectrometry and co-IP data, we conclude that RNase J2 primarily interacts with RNase J1, but exhibits robust interactions with Ldh, RNase Y, and CshA, has weak/transient interactions with DnaK, DnaJ, and FtsZ, and has a questionable interaction with enolase.

### RNase J2 Membrane Association Is Mediated via Multiple Segments of the Protein

**FIGURE 4:**
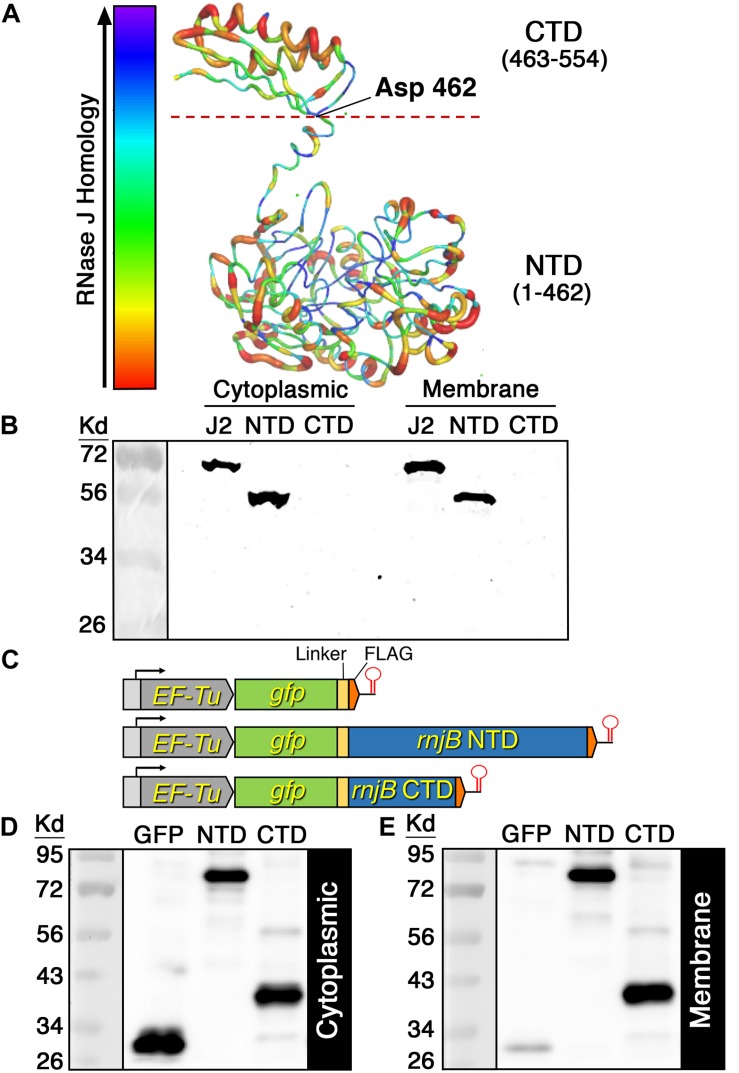
RNase J2 structure model and domain localization. **(A)** The RNase J2 protein structure was predicted using the YASARA WHAT IF “transgenic” homology modeling algorithm. The resulting structure is color-coded to signify the relative sequence conservation between RNase J enzymes. Thin blue regions indicate regions of highest sequence conservation, while thick red regions indicate regions of lowest sequence conservation. The RNase J2 N-terminal domain (NTD) and C-terminal domain (CTD) are indicated. The dashed red line at Asp 462 represents the NTD/CTD split site used for domain localization studies. **(B)** C-terminally FLAG tagged full length RNase J2 (J2), NTD, and CTD were each expressed from a multicopy plasmid. Protein samples were derived from cultures collected at an optical density OD_600_ 0.6. Cytoplasmic and membrane fractions were analyzed by SDS-PAGE and immunoblotted with anti-FLAG antibody. **(C)** Schematic representation of chromosomally encoded GFP fusion protein expression constructs. A total of 20 μg of **(D)** cytoplasmic and **(E)** membrane protein extracts were separated via SDS-PAGE gel and analyzed by western blot using anti-FLAG antibodies. Strains from left to right: FLAG tagged GFP (GFP), FLAG tagged chimeric GFP-NTD (NTD), and FLAG tagged chimeric GFP-CTD (CTD).

One of the primary mechanisms employed by the *E. coli* RNase E to target the cell membrane is via an encoded amphipathic helix (Khemici et al., 2008). Given that RNase J2 is highly abundant in membrane fractions despite its lack of obvious transmembrane segments, we were curious whether it contains putative amphipathic helices or some other structures that might directly target the cell membrane. Using the YASARA WHAT IF “transgenic” homology modeling algorithm, we developed an RNase J2 structural model. The presented model is color coded such that areas in the blue range of the spectrum indicate regions of greatest sequence conservation and therefore highest structural similarity to other RNase J enzymes. Conversely, regions colored in the red range of the spectrum have lower RNase J sequence homology and likely exhibit a higher structural divergence from other RNase J enzymes. As shown in Figure 4A, RNase J2 is comprised of two distinct domain structures referred to as the N-terminal domain (NTD) and C-terminal domain (CTD). The NTD encompasses most of the protein and contains all of the predicted RNA binding sites and catalytic residues. In contrast, the CTD has no predicted roles in RNA metabolism. Overall, we were unable to identify any obvious membrane binding motifs or exposed amphipathic helices within the predicted RNase J2 structure. Therefore, we were curious whether the NTD or CTD might contribute to membrane localization. To test this, we expressed the NTD and CTD on multicopy vectors in *S. mutans* and examined their localization in both the cytoplasmic and membrane fractions. Interestingly, the NTD was abundant in both fractions, indicating that it has an inherent ability to associate with the membrane (Figure 4B). However, we failed to detect the CTD in either the cytoplasmic or membrane fractions, which suggested that it was likely degraded (Figure 4B). To stabilize the CTD, we fused it to the *gfp* ORF and expressed the chimeric *gfp-*CTD ORF as a transcription fusion downstream of the constitutively expressed *EF-Tu* gene (Figure 4C). For comparison, we similarly placed a chimeric *gfp-*NTD ORF and a *gfp* ORF under the transcriptional control of the *EF-Tu* promoter (Figure 4C). In agreement with the previous results, the chimeric GFP-NTD fragment was abundant in both the cytoplasmic and membrane fractions, suggesting the GFP fusion had no impact upon NTD localization (Figures 4D,E). As predicted, the GFP fusion also stabilized the CTD allowing its detection via western blot. Interestingly, the CTD localization pattern mirrored that of the NTD, as similarly abundant signals were present in both fractions (Figures 4D,E). This is in stark contrast to the GFP protein, which was almost exclusively localized in the cytoplasmic fraction (Figures 4D,E). From these results, we conclude that both the NTD and CTD possess independent membrane targeting abilities.

### The RNase J2 CTD Is a Protein–Protein Interaction Domain

**FIGURE 5:**
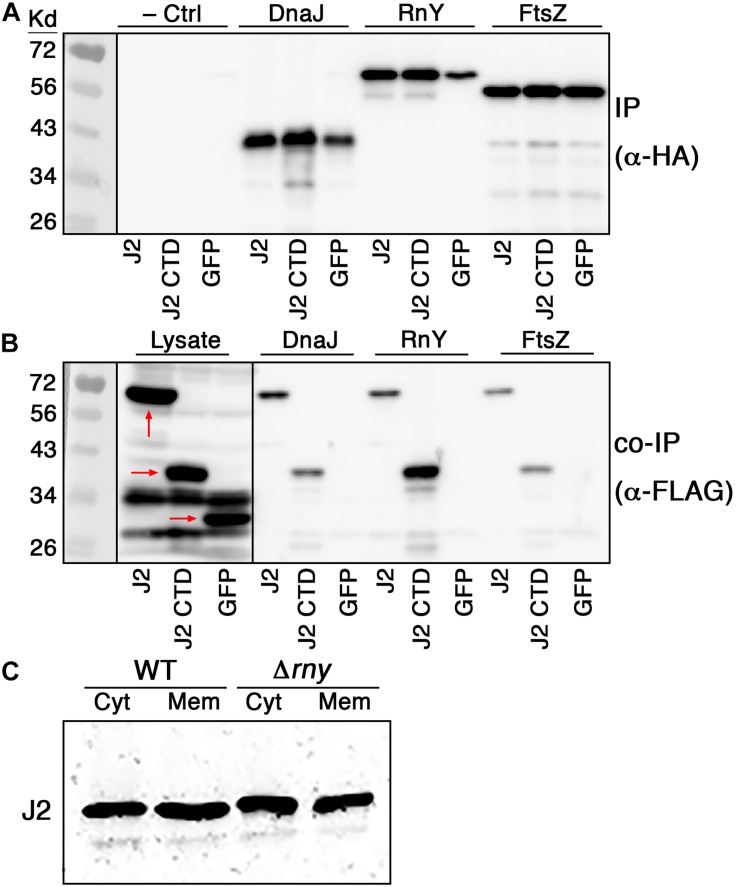
RNase J2 interactions with membrane localized interaction partners. Binary RNase J2 protein interactions were tested in *E. coli* transformed with expression vectors encoding FLAG tagged RNase J2 (J2), chimeric GFP-CTD (J2 CTD), and GFP (GFP) as well as HA tagged DnaJ (DnaJ), RNase Y (RnY), and FtsZ (FtsZ). **(A)** Samples were immunoprecipitated with anti-HA antibodies and then probed with anti-HA antibodies to detect the abundance of DnaJ, RNase Y, and FtsZ. The results in the first three lanes of the top panel are derived from samples solely containing FLAG tagged expression constructs for RNase J2 (J2), chimeric GFP-CTD (J2 CTD), and GFP (GFP) and served as negative controls to assess the specificity of anti-HA immunopurification. **(B)** The first three lanes of the panel are the results obtained after directly immunoblotting lysates of the FLAG tagged expression constructs using anti-FLAG antibodies. These samples serve as molecular weight markers for RNase J2 (J2), chimeric GFP-CTD (J2 CTD), and GFP (GFP) (each indicated by red arrows). The remaining lanes in the panel represent samples that were immunoprecipitated with anti-HA antibodies and then probed with anti-FLAG antibodies to detect the coimmunoprecipitated abundance of RNase J2 (J2), chimeric GFP-CTD (J2 CTD), and GFP (GFP). **(C)** 40 μg of cytoplasmic (Cyt) and membrane (Mem) protein fractions from the parental RNase J2-FLAG (WT) strain and its derivative RNase Y mutant (Δ*rny*) strain were immunoblotted with anti-FLAG antibodies to compare subcellular localization.

Given the surprising ability of the RNase J2 CTD to traffic to the cell membrane without encoding obvious transmembrane segments or amphipathic helices, we were curious whether its membrane localization was dependent upon complex formation with another membrane localized protein, especially RNase Y. RNase Y is the only degradosome-like protein in RNase J-encoding species that contains a transmembrane segment. As such, it has been suggested to serve an analogous role to the *E. coli* RNase E for recruiting other degradosome components to the cell membrane (Lehnik-Habrink et al., 2011, 2012). Therefore, we selected RNase Y and two additional membrane-associated proteins (DnaJ and FtsZ) from our confirmed RNase J2 interacting proteins to test whether they can directly interact with the full-length RNase J2 as well as the RNase J2 CTD. Since co-IP cannot definitively distinguish between direct and indirect protein interactions within protein complexes, we expressed the proteins in *E. coli* to reduce the likelihood of interference from other potential interacting proteins such as RNase J1 and CshA. Also, due to potential protein stability issues, we once again expressed the RNase J2 CTD as a chimeric fusion to GFP. As shown in Figures 5A,B, DnaJ, RNase Y, and FtsZ each formed protein complexes with both the full-length RNase J2 and the chimeric GFP-CTD fusion. No obvious binary interactions were observed with GFP, indicating that the observed CTD interactions were unlikely to be an artifact caused by its fusion to GFP. The RNase Y-CTD interaction was noticeably stronger than the DnaJ- and FtsZ-CTD interactions, which seemed consistent with a role for RNase Y as a scaffold for RNase J2 membrane localization. As a final test, we deleted *rny* in *S. mutans* and examined the localization of RNase J2. Counter to expectations, the Δ*rny* mutation had no obvious impact upon RNase J2 membrane localization (Figure 5C). Thus, protein complex formation between RNase Y and the RNase J2 CTD is at best minimally responsible for localization at the cell membrane. However, the direct interactions observed between the CTD and DnaJ, RnY, and FtsZ indicate that the CTD potentially serves as a protein–protein interaction domain for RNase J2.

### The RNase J2 CTD Is a Common Structural Feature of RNase J Enzymes

**FIGURE 6:**
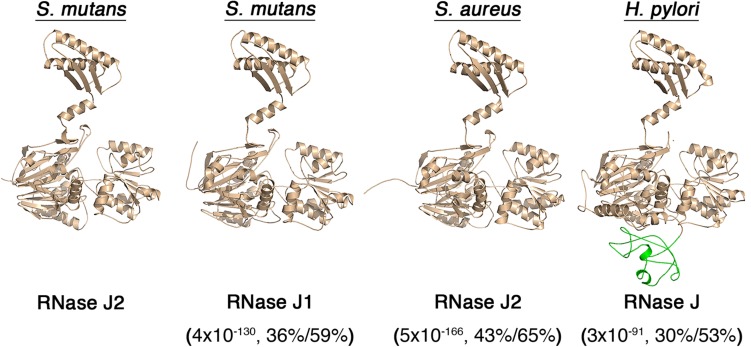
Predicted structures of RNase J paralogs from different organisms. RNase J structural models were constructed using the YASARA WHAT IF “transgenic” homology modeling algorithm. The predicted structures of *S. mutans* RNase J2, *S. mutans* RNase J1, *S. aureus* RNase J2, and *Helicobacter pylori* RNase J are presented. An additional RNase J domain unique to *H. pylori* is illustrated in green. The values listed beneath each enzyme indicate the BLASTP *e*-value,% identity, and % similarity to the *S. mutans* RNase J2.

The overall sequence conservation of RNase J enzymes is quite high, even among phylogenetically distant organisms (Supplementary Figure S1). However, sequence homology is not uniform across the lengths of the proteins, as sequence conservation drops considerably in the C-terminal portions of RNase J enzymes (Supplementary Figure S1). Despite this, the *B. subtilis* and *Thermus thermophilus* RNase J1 crystal structures both reveal CTDs bearing a strong resemblance to the predicted CTD of the *S. mutans* RNase J2 (Li de la Sierra-Gallay et al., 2008; Newman et al., 2011). Therefore, we created several additional structural models of other RNase J enzymes to determine whether the CTD is likely to be a conserved structural feature. Indeed, this is what we observed. RNase J1, RNase J2, and RNase J all exhibit similar architectures consisting of a large NTD connected to a linker α-helix followed by a CTD consisting of a 3-stranded β-sheet and two α-helices (Figure 6). RNase J from *H. pylori* also contains an additional unstructured region within the NTD that appears unique to this protein, but it otherwise exhibits a highly analogous overall architecture with other RNase J enzymes (Figure 6). Thus, the RNase J CTD is apparently a highly conserved structural feature of these enzymes. While the CTD likely has a conserved functional role as an RNase J dimerization interface (Li de la Sierra-Gallay et al., 2008; Newman et al., 2011), this structure may serve an additional role in RNase J enzymes as a protein–protein interaction domain similar to that of the *S. mutans* RNase J2.

## Discussion

**FIGURE 7:**
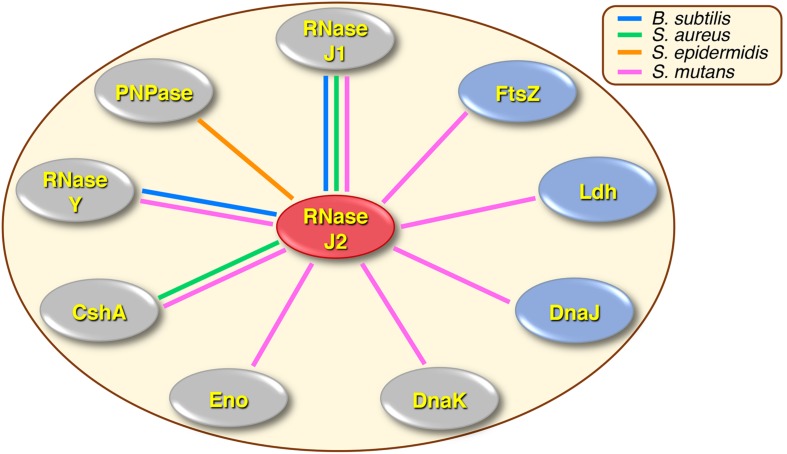
Summary of RNase J2 protein interactions in different organisms. Colored lines drawn between RNase J2 and other proteins indicate experimentally determined protein interactions. Line colors signify the organisms in which the studies were conducted: blue lines indicate *B. subtilis*, green lines indicate *S. aureus*, orange lines indicate *S. epidermidis*, and pink lines indicate *S. mutans*. Protein interactions implicated in degradosome or degradosome-like complexes are shown in gray ovals, while novel RNase J2 interactions are illustrated in blue ovals.

A summary of our current knowledge of the RNase J2 protein interactome is presented in Figure 7. In *B. subtilis*, the function of RNase J2 has remained somewhat enigmatic, as RNase J2 mutants exhibit minimal phenotypes and its reported protein interactions have thus far been limited to RNases J1 and Y (Mathy et al., 2010; Lehnik-Habrink et al., 2011; Figaro et al., 2013). Conversely, in other species like the streptococci and staphylococci, RNase J2 is a critical pleiotropic regulator that controls the expression of a highly diverse regulon. Accordingly, an RNase J2 mutation is lethal in the Group A *Streptococcus*, while in *S. mutans* it triggers a severe growth deficiency even greater than that of an RNase J1 mutation (Bugrysheva and Scott, 2010; Chen et al., 2015). Despite this, RNase J2 has remained largely understudied relative to its paralogous enzyme RNase J1.

### A Degradosome in *S. mutans*?

Whether RNase J-encoding organisms primarily assemble degradosome-like complexes analogous to those of *E. coli* is still the subject of active debate in the field. A variety of RNase J1 and Y two-hybrid studies in *B. subtilis* and *S. aureus* have identified multiple protein interactions that are reminiscent of those found in the *E. coli* degradosome (Commichau et al., 2009; Mathy et al., 2010; Lehnik-Habrink et al., 2011; Roux et al., 2011; Salvo et al., 2016). However, two-hybrid protein interactions are also measured outside of their native context, so the relative strengths and abundances of the RNase J1 and Y degradosome-like interactions are still somewhat obscure. In addition, the *E. coli* degradosome is characterized by its membrane localization mediated by RNase E (Liou et al., 2001; Khemici et al., 2008), while RNase J-encoding species also encode the membrane associated endoribonuclease RNase Y, which was proposed to serve as the functional equivalent of RNase E (Lehnik-Habrink et al., 2011). However, the enzymes proposed to comprise RNase J degradosome-like complexes do not exhibit similarly strict membrane association, as might be expected if they were largely confined to a degradosome (Lehnik-Habrink et al., 2011; Cascante-Estepa et al., 2016). Likewise, previous attempts to purify an intact RNA degradosome from *B. subtilis* have thus far proven unsuccessful (Lehnik-Habrink et al., 2011; Cascante-Estepa et al., 2016). In our study of the *S. mutans* RNase J2, we detected protein interactions with components of the proposed RNase J degradosome-like complex, such as RNase J1, RNase Y, RNA helicase, and possibly enolase (Figures 2A,B, 3A,B). RNase J2 also binds to the heat shock protein DnaK (Figures 2C,D, 3A,B), which is a component of the *E. coli* degradosome (Miczak et al., 1996), but has not been previously reported for RNase J degradosome-like complexes. While these results may seem supportive of a degradosome, most of these interactions were considerably weaker than the RNase J1-J2 interaction. Since our studies were performed using *in vivo* crosslinking prior to cell lysis, one would expect the different degradosome proteins to yield more comparable co-IP signal intensities if indeed they were primarily purified from stable complexes similar to what we observed for RNase J1/J2 (Figures 2A–D). Furthermore, we failed to confirm RNase J2 interactions with phosphofructokinase or PNPase (Figures 2A,B), which have both been identified in RNase J1 and Y protein interaction studies (Commichau et al., 2009; Lehnik-Habrink et al., 2011; Roux et al., 2011) and should have been detected in our studies if they were components of a stable degradosome-like complex with RNase J2. Certainly, we cannot exclude the possibility that the observed co-IP results are simply a function of the stoichiometry of proteins found within RNase J2 complexes. However, it seems unlikely that nearly all of these protein interactions would exhibit a stoichiometry favoring a substantial overrepresentation of RNase J2. In addition to the aforementioned degradosome-like interactions, we also identified several novel RNase J2 protein interactions that have not been previously reported in interactome studies of RNases E, J, or Y, such as the heat shock protein DnaJ, the cell division GTPase FtsZ, and the central metabolic enzyme Ldh. The RNase J2-Ldh interaction was among the strongest we detected in both our mass spectrometry proteomic data and in our co-IP studies (Figures 2C,D, 3A,B and Supplementary Table S3). Interestingly, this interaction was also heavily biased for the cytoplasmic fraction (Figures 2C,D, 3A,B and Supplementary Table S3), which is counter to the current understanding of degradosome interactions. RNase J2 interactions with RNase Y, DnaJ, and FtsZ were further tested in *E. coli* and confirmed to exhibit binary interactions, suggesting these proteins can interact directly with RNase J2 (Figures 5A,B). Given the large number of untested candidate RNase J2 interactions remaining in the mass spectrometry data (Supplementary Table S3), it is reasonable to assume that RNase J2 likely exhibits a number of additional novel protein interactions. Such a result would also be consistent with the pleiotropic regulatory role of RNase J2 in *S. mutans* and other species. Overall, our current RNase J2 interactome results are inconsistent with the existence of a stable degradosome in *S. mutans*, unless degradosome interactions only account for a minority of the total protein interactions with RNase J2. In agreement with previous studies in *B. subtilis*, our results do suggest that the vast majority of RNase J2 is found within stable RNase J1/J2 complexes located in both the cytoplasm and at the cell membrane. We suspect that these heteromeric RNase J1/J2 complexes probably form transient interactions with many different proteins, some of which likely resemble the *E. coli* degradosome. It is also possible that RNase J2 (and J1) participates in unrecognized moonlighting functions facilitated by these diverse protein interactions.

### The RNase J CTD

As previously mentioned, the CTD of RNase J1 was noted in two separate RNase J1 crystal structures and an analogous CTD is even present in RNase E (Li de la Sierra-Gallay et al., 2008; Newman et al., 2011). Our structural models of RNases J1, J2, and J all predicted strikingly similar CTDs among the RNase J enzymes from both Gram positive and Gram negative species (Figure 6). This was initially surprising because the C-terminal regions of RNase J enzymes also exhibit the lowest sequence conservation (Supplementary Figure S1). This suggests there is likely to be a selective pressure to maintain this structure, even though its primary sequence has diverged more than other areas of the protein. Structural conservation of the CTD is likely to be at least partially attributable to its role as a major portion of the RNase J dimer interface (Li de la Sierra-Gallay et al., 2008; Newman et al., 2011). However, our results suggest that the CTD might serve an additional unrecognized function as a major site of RNase J protein–protein interactions. At a minimum, the *S. mutans* RNase J2 CTD likely interacts directly with DnaJ, RNase Y, and FtsZ (Figure 5B). This may be functionally analogous to the *E. coli* RNase E, which also employs its C-terminal region for a large number of protein interactions, including assembly of the degradosome (Vanzo et al., 1998; Taghbalout and Rothfield, 2007; Dominguez-Malfavon et al., 2013). We also determined that the RNase J2 CTD has an inherent ability to target the cell membrane (Figure 4E). Presently, it is unclear whether this ability is mediated by protein–protein interactions with a membrane-associated protein or if the CTD has an intrinsic affinity for the membrane. However, we confirmed that RNase Y is not essential for RNase J2 membrane localization (Figure 5C), nor is the CTD (Figure 4E).

## Experimental Procedures

### Bacterial Strains and Culture Conditions

*Streptococcus mutans* UA159 and *E. coli* strain BL21 served as wild-type/parental strains used in this study. *S. mutans* was cultured in a 5% CO_2_ atmosphere at 37°C in Todd–Hewitt medium (Difco) supplemented with 0.3% (w/v) yeast extract (THYE). For antibiotic selection, 10 μg ml^–1^ erythromycin or 1000 μg ml^–1^ spectinomycin was used for *S. mutans* and 50 μg ml^–1^ kanamycin was used for *E. coli*. For experiments requiring xylose induction, cultures were supplemented with 1% (wt/vol) xylose. The *S. mutans* competence-stimulating peptide (CSP) (SGSLSTFFRLFNRSFTQALGK) was synthesized by Anaspec and supplemented in cultures at a concentration of 1 μg ml^–1^ for transformation reactions. For counterselection, THYE plates were supplemented with 0.4% (w/v) p-chlorophenylalanine (4-CP, Sigma).

### DNA Manipulation and Strain Construction

The 3x FLAG epitope (DYKDHDGDYKDHDIDYKDDDDK), FLAG epitope (DYKDDDDK), or HA epitope (YPYDVPDYA) were added to the C- or N-termini of specific proteins using either a markerless mutagenesis strategy (Xie et al., 2011; Zhang et al., 2017) or via allelic replacement with an antibiotic cassette. Transformation reactions were performed as follows. Bacteria were diluted 1:20 from overnight cultures and grown to an optical density of OD_600_ ∼0.2 in THYE before adding 500 ng ml^–1^ transforming DNA and 1 μg ml^–1^ CSP. The cultures were subsequently incubated for an additional 2 h and then plated on selective media. The strains, plasmids, and primers used in this study are listed in Supplementary Tables S1, S2. Phusion DNA polymerase (NEB) was used to amplify individual PCR fragments, while AccuPrime Polymerase (Invitrogen) was used for overlap extension PCR (OE-PCR). All constructs and/or genetic modifications were verified by PCR and sequencing.

#### FLAG and HA Epitope Tagging

A previously described 2-step markerless mutagenesis strategy (Xie et al., 2011)was used to insert DNA sequences encoding FLAG or HA epitopes onto the *S. mutans* chromosome immediately upstream of the stop codons of *rnjB*, *rnjA*, *cshA*, *pnp*, *rny*, *dnaK*, *ftsZ*, *ldh*, *infB*, *rpoB*, and SMU_965. To generate the first construct required for the RNase J2-FLAG strain, a ∼1 kb fragment of the *rnjB* ORF and an adjacent ∼1 kb fragment downstream of the *rnjB* ORF were both amplified from UA159 genomic DNA with the primer pairs J2upF1/J2upR1 and J2dnF1/J2dnR1, respectively. The IFDC3 counterselection cassette was PCR amplified with the primer pair IFDC3F/IFDC3R (Zhang et al., 2017). Both ∼1 kb PCR amplicons contain terminal complementarity with the IFDC3 cassette, which facilitated their assembly into a single PCR amplicon via OE-PCR using the primer pair J2upF1/J2dnR1. The resulting OE-PCR amplicon was transformed into *S. mutans* UA159 and selected on agar plates supplemented with erythromycin. To generate the second construct required for the RNase J2-FLAG strain, a ∼1 kb fragment of the *rnjB* ORF lacking its stop codon was amplified from UA159 genomic DNA with the primers J2upF1/J2upR2. The resulting PCR amplicon was reamplified with the primer pair J2upF1/J2upR3 to add the remaining sequence of a 3x FLAG ORF onto the 3′ of the amplicon. An adjacent ∼1 kb fragment downstream of the *rnjB* ORF was amplified with the primers J2dnF2/J2dnR1. Both amplicons contain terminal complementarity, which facilitated their assembly into a single PCR amplicon via OE-PCR using the primer pair J2upF1/J2dnR1. The resulting OE-PCR amplicon was transformed into the intermediate strain generated in the previous step and selected on agar plates supplemented with 4-CP to generate the markerless RNase J2-FLAG strain J2F. To generate the first construct required for the RNase J1-HA strain, a ∼1 kb fragment of the *rnjA* ORF and an adjacent ∼1 kb fragment downstream of the *rnjA* ORF were both amplified from UA159 genomic DNA with the primer pairs J1upF1/J1upR1 and J1dnF1/J1dnR1, respectively. The IFDC3 counterselection cassette was PCR amplified with the primer pair IFDC3F/IFDC3R. Both ∼1 kb PCR amplicons contain terminal complementarity with the IFDC3 cassette, which facilitated their assembly into a single PCR amplicon via OE-PCR using the primer pair J1upF1/J1dnR1. The resulting OE-PCR amplicon was transformed into strain J2F and selected on agar plates supplemented with erythromycin. To generate the second construct required for the RNase J1-HA strain, a ∼1 kb fragment of the *rnjB* ORF lacking its stop codon was amplified from UA159 genomic DNA with the primers J1upF1/J1upR2, which added an HA epitope sequence onto the 3′ of the amplicon. An adjacent ∼1 kb fragment downstream of *rnjA* ORF was amplified with the primers J1dnF2/J1dnR1. Both amplicons contain terminal complementarity, which facilitated their assembly into a single PCR amplicon via OE-PCR using the primer pair J1upF1/J1dnR1. The resulting OE-PCR amplicon was transformed into the intermediate strain generated in the previous step and selected on agar plates supplemented with 4-CP to generate the markerless RNase J1-HA strain J1HA. The same assembly strategy used for strain J1HA was employed to create the following HA epitope tagged strains: CshAHA, PnpHA RnyHA, DnaKHA, FtsZHA, LdhHA, InfBHA, RpoBHA, and 965HA. The only modification to the above strategy was for strain PnpHA, which used the primer pair IFDC3F/IFDC3Rrbs to amplify the IFDC3 fragment. A slightly different approach was employed to add HA epitope tags onto PfkA and Eno. To modify these two proteins, we employed Direct Repeat-Mediated Cloning-Independent Markerless Mutagenesis (DR-CIMM) (Zhang et al., 2017). For this approach, two constructs are required, but only a single transformation step is necessary. To generate the first constructs required for the PfkA-HA or Eno-HA strains, ∼1 kb fragments including the *pfkA* or *eno* ORFs without their stop codons were amplified from UA159 genomic DNA with the primers PfkupF1/PfkupR1 or EnoupF1/EnoupR1. Both primer sets added an HA epitope sequence onto the 3′ ends of the amplicons followed by sequence complementary to the IFDC3 cassette. The IFDC3 cassette was PCR amplified with the primer pair IFDC3F/IFDC3R. The *pfkA* and *eno* amplicons were ligated to the IFDC3 amplicon via OE-PCR reactions with the primer pairs PfkupF1/IFDC3R or EnoupF1/IFDC3R. To generate the second construct, ∼200 bp amplicons homologous to the 3′ ends of the *pfkA* and *eno* ORFs were PCR amplified with the primers PfkupF2/PfkupR2 or EnoupF2/EnoupR2. Both primer sets added an HA epitope sequence onto the 3′ end of the fragments as well as IFDC3 complementarity onto the 5′ end of the fragments. ∼1 kb homologous fragments downstream of the *pfkA* or *eno* stop codons were amplified with the primers PfkdnF1/PfkdnR1 or EnodnF1/EnodnR1. The three amplicons were mixed and assembled via OE-PCR with the primers IFDC3F/PfkdnR1 or IFDC3F/EnodnR1. The two OE-PCR amplicons were mixed and simultaneously transformed into strain J2F and selected on agar plates containing erythromycin. Antibiotic resistant transformants were subsequently subcultured without antibiotic and spotted onto 4-CP agar plates to generate the strains PfkAHA and EnoHA. The DnaJ and FtsA proteins were modified with N-terminal HA epitopes using a marked allelic replacement strategy. ∼1 kb homologous fragments upstream of the *dnaJ* and *ftsZ* ORFs were amplified from UA159 genomic DNA with the primer pairs DJupF1/DJupR1 or FAupF1/FAupR1. A second homologous fragment ∼1 kb downstream of the *dnaJ* or *ftsA* start codons was amplified with the primers DJdnF1/DJdnR1 or FAdnF1/FAdnR1, which also added HA epitope sequences onto the 5′ ends of the amplicons. These amplicons were reamplified with the primer pairs DJdnF2/DJdnR1 or FAdnF2/FAdnR1 to add erythromycin cassette complementarity to the final downstream fragments. The erythromycin cassette was amplified with the primers ErmF1/ErmRDJ or ErmF1/ErmRFA. The three amplicons were mixed and assembled via OE-PCR with the primer pairs DJupF1/DJdnR1 or FAupF1/FAdnR1 to generate the final constructs. The resulting OE-PCR amplicons were transformed to strain J2F and selected on agar plates containing erythromycin to generate the final strains DnaJHA and FtsAHA.

#### Construction of Chromosomal GFP Fusion Proteins

To constitutively express chimeric GFP fusion proteins from the *EF-Tu* promoter, we employed a marked allelic replacement strategy to insert *gfp* fusion constructs onto the *S. mutans* chromosome between the *EF-Tu* ORF stop codon and the *EF-Tu* transcription terminator. The mNeonGreen *gfp* ORF (Shaner et al., 2013) was codon optimized for expression in *S. mutans* and synthesized by Integrated DNA Technologies. To generate the *gfp*-3x FLAG construct, we amplified the 3x FLAG sequence from plasmid pVAsf using the primers ng3F/ng4R. A fragment encoding GFP and the linker peptide GGGGS was amplified from strain UA159G using the primer pairs ng1F/ng2R, while the erythromycin resistance cassette was amplified from strain UA159G using the primers ng5F/ng6R. The three PCR amplicons each contained terminal complementarity which facilitated their assembly via OE-PCR. The OE-PCR amplicon was transformed into strain UA159 and selected on agar plates containing erythromycin to generate the strain UA159GF. To generate the chimeric GFP-NTD-3x FLAG construct, we amplified a fragment encoding a C-terminal 3x FLAG tagged NTD ORF from plasmid pVAJ2-1 using the primers ng9F/ng4R. The *gfp* ORF and linker peptide GGGGS sequence was amplified from strain UA159G using the primers ng1F/ng7R, while the erythromycin resistance cassette was amplified from strain UA159G using the primers ng5F/ng6R. The three PCR amplicons each contained terminal complementarity which facilitated their assembly via OE-PCR. The OE-PCR amplicon was transformed into strain UA159 and selected on agar plates containing erythromycin to generate the strain J2NTDGF. To generate the chimeric GFP-CTD-3x FLAG construct, we amplified a fragment encoding a C-terminal 3x FLAG tagged CTD ORF from plasmid pVAJ2-2 using the primers ng8F/ng4R. A fragment encoding GFP and the linker peptide GGGGS was amplified from strain UA159G using the primers ng1F/ng7R, while the erythromycin resistance cassette was amplified from strain UA159G using the primers ng5F/ng6R. The three PCR amplicons each contained terminal complementarity which facilitated their assembly via OE-PCR. The OE-PCR amplicon was transformed into strain UA159 and selected on agar plates containing erythromycin to generate the strain J2CTDGF.

#### Construction of *S. mutans* and *E. coli* Protein Expression Vectors

All plasmids were assembled via a Gibson cloning strategy (Gibson, 2011). To express a FLAG tagged FtsH in *S. mutans*, the *ftsH* ORF was amplified from wild-type *S. mutans* strain UA140 with the primer pair ftsHF1/ftsHR2, which adds a 5′ FLAG ORF onto *ftsH*. The resulting amplicon was reamplified with the primer pair ftsHF3/ftsHR2 to add additional DNA used for subsequent Gibson assembly. Linear pZX9 vector backbone was amplified with the primer pair pZX9F/pZX9R (Xie et al., 2013). The two amplicons were mixed, assembled according to the published Gibson assembly protocol, and then transformed into *S. mutans* UA140 to obtain the plasmid pZX9h. The plasmid pZX9h was transformed into strain UA159GF and selected on agar plates supplemented with spectinomycin to obtain strain UA159GFH. To express a 3x FLAG tagged GFP in *S. mutans*, a plasmid backbone containing the 3x FLAG sequence was amplified via inverse PCR of the plasmid pVAJ2-3 using the primers sf1F/sf2R. The *gfp* ORF was amplified with the primers sf3F/sf4R, which added complementary sequences to the linearized plasmid. The two amplicons were assembled according to the published Gibson assembly protocol, transformed directly into *S. mutans*, and then selected on agar plates containing erythromycin to obtain the plasmid pVAsf within strain UA159SG. To express the *rnjB* CTD in *S. mutans*, the CTD ORF with 3x FLAG sequence was amplified from strain J2F genomic DNA with the primers su3F/su4R. A plasmid backbone was amplified via inverse PCR of the plasmid pDL278e using primers su1F/su2R, which added complementary sequences to the amplified CTD fragment. The two amplicons were assembled according to the published Gibson assembly protocol, transformed directly into *S. mutans*, and then selected on agar plates containing erythromycin to obtain the plasmid pVAJ2-1 within strain UA159J2CTD. To express the *rnjB* NTD in *S. mutans*, a plasmid backbone containing the 3x FLAG sequence was amplified via inverse PCR of the plasmid pVAJ2-1 using the primers su5F/su6R. The NTD ORF was amplified from strain UA159 genomic DNA with the primers su7F/su8R. The two amplicons were assembled according to the published Gibson assembly protocol, transformed directly into *S. mutans*, and then selected on agar plates containing erythromycin to obtain the plasmid pVAJ2-2 within strain UA159J2NTD. To perform RNase J2 co-IP studies in *E. coli*, we cloned the *rnjB* ORF, *gfp-*CTD ORF, and *gfp* ORF into the pET29b vector together with the *rny*, *dnaJ*, and *ftsZ* ORFs for co-expression. For plasmid pJ2F, an ORF encoding a 3x FLAG tagged *rnjB* was amplified from strain J2F using the primers p3F/p4R, while the vector backbone was amplified via inverse PCR of pET29b using the primers p1F/p2R. The two amplicons were assembled according to the published Gibson assembly protocol, transformed directly into *E. coli*, and then selected on agar plates containing kanamycin. For plasmid pJ1HJ2F, an ORF encoding a 3x FLAG tagged *rnjB* was amplified from strain J2F using the primers p5F/p4R, while the vector backbone was amplified via inverse PCR of pET29b-J1J2 (unpublished) using the primers p1F/p6R, which added an HA epitope sequence onto *rnjA* in the vector. The two amplicons were assembled according to the published Gibson assembly protocol, transformed directly into *E. coli*, and then selected on agar plates containing kanamycin. The plasmid pJ1HJ2F was then used as a template to amplify via inverse PCR with the primers p7F/p8R to generate the remaining constructs, including pDnaJHJ2F, pFtsZHJ2F and pRnYHJ2F. Inserts encoding HA tagged DnaJ, FtsZ, and RnY were amplified from the genomic DNA of strains DnaJHA, FtsZHA, and RnYHA using the primer pairs p11F/p12R, p15F/p10R, and p17F/p18R, respectively. For each of these constructs, the vector and insert amplicons were assembled according to the published Gibson assembly protocol, transformed directly into *E. coli*, and then selected on agar plates containing kanamycin. To perform co-IP of the RNase J2 CTD with DnaJ, FtsZ, and RnY in *E. coli*, the plasmids pDnaJHJ2T, pFtsZHJ2T, and pRnYHJ2T were constructed. The primer pairs p19F/p20R, p21F/p20R, and p22F/p20R were used to amplify inserts encoding GFP-CTD-3x FLAG from strain J2CTDGF genomic DNA. The primer pairs p1F/p23R, p1F/p24R, and p1F/p25R were used to amplify the plasmids pFtsZHJ2F, pDnaJHJ2F, and pRnYHJ2F via inverse PCR, which encode HA tagged FtsZ, DnaJ, and RnY, respectively. A control vector pJ2T, expressing only the GFP-CTD-3x FLAG, was also constructed. An insert encoding GFP-CTD-3x FLAG was amplified from the genomic DNA of strain J2CTDGF using the primers p26F/p27R, while the plasmid pJ2F was amplified using the primers p1F/p28R. The two amplicons were assembled according to the published Gibson assembly protocol, transformed directly into *E. coli*, and then selected on agar plates containing kanamycin. To perform co-IP of GFP with DnaJ, FtsZ, and RnY in *E. coli*, we constructed the plasmids pDnaJHG, pFtsZHG, and pRnYHG. The primer pairs p19F/p20R, p21F/p20R, and p22F/p20R were used to amplify inserts encoding GFP-3x FLAG from strain UA159GF genomic DNA. The primer pairs p1F/p23R, p1F/p24R, and p1F/p25R were used to amplify the plasmids pFtsZHJ2F, pDnaJHJ2F, and pRnYHJ2F via inverse PCR, which encode HA tagged FtsZ, DnaJ, and RnY, respectively. A control vector pG, expressing only the GFP-3x FLAG, was also constructed. An insert encoding a GFP-3x FLAG was amplified from the genomic DNA of strain UA159GF using the primers p26F/p27R, while the plasmid pJ2F was amplified using the primers p1F/p28R. The two amplicons were assembled according to the published Gibson assembly protocol, transformed directly into *E. coli*, and then selected on agar plates containing kanamycin.

### *In vivo* Crosslinking and Cellular Fractionation of *S. mutans*

The *in vivo* crosslinking protocol was modified slightly from a previous description (Vasilescu et al., 2004). Firstly, 100 ml cultures were grown to an optical density of OD_600_ of 0.6 in THYE broth. Cultures were centrifuged at 3,220 × *g* for 15 min at 4°C, the resulting cell pellets were resuspended in 20 ml PBS containing 1% (v/v) formaldehyde, and finally incubated at room temperature for 20 min with gentle agitation. Afterward, glycine was added to the solution at a final concentration of 0.1 M at room temperature for 5 min with gentle agitation to quench the reaction. The suspension was centrifuged for 15 min at 3,220 × *g* at 4°C and the resulting pellet was washed twice with an equal volume of PBS. Cytoplasmic and membrane fractionation were performed as previously described (Baev et al., 1999) with minor modifications. Briefly, crosslinked cell pellets were resuspended in 1 ml buffer A [150 mM NaCl, 10% (v/v) Glycerol, and 100 mM Na-phosphate buffer (pH 7.0)] containing 0.2 mM phenylmethanesulfonyl fluoride (PMSF) and 1 mM benzamidine. Cells were lysed in an Omni Bead Ruptor 24 for 12 cycles at 5 m/s for 20 s. The resulting lysates were clarified by centrifuging at 16,000 × *g* for 15 min at 4°C. To separate the cytoplasmic and membrane fractions, supernatants were transferred into 5 ml ultracentrifuge tubes (Beckman) and centrifuged at 105,000 × *g* for 1.5 h at 4°C in an ultracentrifuge (Beckman L8-80M, 50.4 Ti). After centrifugation, the supernatants were carefully removed and transferred to 1.5 ml tubes to serve as the cytoplasmic fractions. The remaining protein pellets were resuspended in 500 μl of buffer A containing 1% Triton X-100 and then solubilized overnight at 4°C with gentle agitation on a rotator. The next day, the solutions were centrifuged at 16,000 × *g* for 15 min at 4°C before collecting the supernatants to serve as the membrane fractions. Protein concentrations were determined using the Bradford assay (Bio-Rad) according to the manufacturer’s protocol. Bovine serum albumin served as the standard.

### Expression and Coimmunoprecipitation of Recombinant Proteins From *E. coli*

*Escherichia coli* strain BL21 harboring expression vectors was cultured overnight at 37°C in LB medium containing 50 μg ml^–1^ kanamycin. Bacteria were then diluted 1:20 in 100 ml fresh LB medium + 50 μg ml^–1^ kanamycin until an optical density of OD_600_ = 0.8. Next, 0.1 mM IPTG was added to the cultures and further incubated at 16°C for 16 h. Cells were pelleted by centrifuging at 3,220 × *g* for 15 min at 4°C, then washed twice with PBS, and finally resuspended in 2 ml of PBS. Cells were sonicated for 10 min (10 s “on” and 15 s “off”) on ice followed by centrifugation at 16,000 × *g* for 15 min at 4°C (Eppendorf, 5424R centrifuge) to recover supernatants. Protein concentrations were determined using the Bradford assay (Bio-Rad) and 1 mg of protein was immunoprecipitated using anti-FLAGM2 Affinity Gel (Sigma) (Gerace and Moazed, 2015) or monoclonal Anti-HA-Agarose (Sigma) (Tomomori-Sato et al., 2013) according to the manufacturer’s protocol.

### Immunoprecipitation and Mass Spectrometry

One mg of total protein was immunoprecipitated using anti-FLAGM2 Affinity Gel (Sigma) according to the manufacturer’s protocol. 3x FLAG Peptide (Sigma) with a working concentration of 100 μg ml^–1^ was used for the competitive elution of 3x FLAG tagged RNase J2. Eluates were loaded into polyacrylamide gels and electrophoresed for 5 min before digesting in gel (Rosenfeld et al., 1992) using Trypsin Gold (Promega) and ProteaseMAXTM Surfactant (Promega) according to the manufacturer’s protocol. The tryptic peptides were extracted, dried in a vacuum dryer, resuspended in 15 μl 0.1% formic acid, and lastly analyzed by LC/MS using a Thermo Scientific Orbitrap Fusion mass spectrometer (Senko et al., 2013).

### *S. mutans* Coimmunoprecipitation

One mg of total cytoplasmic or membrane protein extract was immunoprecipitated using anti-FLAGM2 Affinity Gel (Sigma) or monoclonal Anti-HA-Agarose (Sigma) according to the manufacturer’s protocol. Proteins were eluted by adding 50 μl 1% (w/v) SDS and vortexing for 5 min. Equal volumes of the eluted fractions were separated by SDS-PAGE and then transferred to nitrocellulose membranes (GE Healthcare). Nitrocellulose membranes were blocked overnight at 4°C with 5% (w/v) non-fat dry milk diluted in TBST buffer (0.1% Tween 20 in Tris-buffered saline, pH 7.4). Blots were then incubated 1 h with primary antibodies diluted 1:1000 in 5% milk-TBST at room temperature. Secondary antibodies were diluted 1:5000 in 5% milk-TBST and incubated with the blots for 1 h at room temperature. The blots were detected using the ChemiGlow West Chemiluminescence Substrate Kit (Proteinsimple). Primary anti-FLAG and anti-HA antibodies were purchased from Invitrogen, while horseradish peroxidase (HRP)-conjugated secondary antibodies were purchased from Thermo Fisher Scientific. All co-IP experiments were performed with a minimum of three biological replicates.

### RNase J Structure Modeling

RNase J homology models were constructed using the YASARA WHAT IF “transgenic” homology modeling algorithm. The algorithm first conducted a BLASTP search against the UniProt database to identify homologous structures in the Protein Data Bank (PDB). The resulting alignments were ranked and refined through structural considerations to develop an array of homology models. Loops were modeled through a BLAST search against the PDB and then alignments were scored and optimized. Side chain rotameric states were calculated via backbone-dependent probabilities and optimized through molecular dynamics and knowledge-based force fields. The resulting models were optimized for hydrogen bonding and then refined through molecular dynamics simulations. The models were ranked, and for each model, a residue specific quality graph is calculated. A final hybrid model is then developed through an iterative process, replacing poorly scoring regions in the best model with the corresponding regions from other models.

## Data Availability

All datasets generated for this study are included in the manuscript and/or the Supplementary Files.

## Author Contributions

RM, JK, and JM made major contributions to the conception and/or design of the study, and contributed to the writing and editing of the manuscript. RM, PS, and ZZ contributed to the acquisition, analysis, and interpretation of the data.

## Conflict of Interest Statement

The authors declare that the research was conducted in the absence of any commercial or financial relationships that could be construed as a potential conflict of interest.
