# Supplementary material for: Examining the Protein Interactome and Subcellular Localization of RNase J2 Complexes in Streptococcus mutans
Source: Front Microbiol. 2019 Sep 18;10:2150. doi: 10.3389/fmicb.2019.02150 (PMC6759994; doi:10.3389/fmicb.2019.02150)
Supplement: Supplementary file 1 [file Data_Sheet_1.docx]

**Table S1. Strains and plasmids used in this study.**

| Strains/Plasmids | Description^*^ | Reference |
| --- | --- | --- |
| *Strains* |  |  |
| UA140 | WT *Streptococcus mutans* | (Qi *et al.*, 2001) |
| UA159 | WT *Streptococcus mutans* | (Ajdic *et al.*, 2002) |
| T260S | UA140::IFDC3^pheS A314G/T260S^, Em^r^, | (Zhang *et al.*, 2017) |
| J2F | UA159::*rnjB-*3x FLAG | This study |
| J1HA | J2F::*rnjA-*HA | This study |
| CshAHA | J2F::*cshA-*HA | This study |
| EnoHA | J2F::*eno-*HA | This study |
| PfkAHA | J2F::*pfkA-*HA | This study |
| PnpHA | J2F::­*pnp-*HA | This study |
| RnyHA | J2F::*rny-*HA | This study |
| DnaKHA | J2F::*dnaK-*HA | This study |
| DnaJHA | J2F::HA-*dnaJ* | This study |
| FtsZHA | J2F::*ftsZ-*HA | This study |
| FtsAHA | J2F::HA-*ftsA* | This study |
| LdhHA | J2F::*ldh-*HA | This study |
| InfBHA | J2F::*infB-*HA | This study |
| RpoBHA | J2F::*rpoB-*HA | This study |
| 965HA | J2F::SMU_965-HA | This study |
| UA159SG | UA159::pVAsf, Em^r^ | This study |
| UA159J2CTD | UA159::pVAJ2-1, Em^r^ | This study |
| UA159J2NTD | UA159::pVAJ2-2, Em^r^ | This study |
| UA159G | UA159::(*EF-Tu-gfp* [NeonGreen]), Em^r^ | Unpublished |
| UA159GF | UA159::(*EF-Tu-gfp* [NeonGreen]*-*3x FLAG), Em^r^ | This study |
| J2NTDGF | UA159::(*EF-Tu-gfp* [NeonGreen]*-rnjB* [NTD]-3x FLAG), Em^r^ | This study |
| J2CTDGF | UA159::(*EF-Tu-gfp* [NeonGreen]*-rnjB* [CTD]-3x FLAG), Em^r^ | This study |
| UA159GFH | UA159GF::pZX9h, Em^r^, Spec^r^ | This study |
| *Plasmids* |  |  |
| pDL278e | *E. coli*-*Streptococcus* shuttle vector, Em^r^ | Unpublished |
| pZX9 | pVA380*::gyrAP::xylR::ldhP::xylAO::luc,* Spec^r^ | (Xie *et al.*, 2013) |
| pZX9h | pVA380*::gyrAP::xylR::ldhP::xylAO::*FLAG-*ftsH,* Spec^r^ | This study |
| pVAsf | pVA380::(*gfp* [Superfolder]-3x FLAG), Em^r^ | This study |
| pVAJ2-1 | pVA380::(*rnjB* [CTD]-3x FLAG), Em^r^ | This study |
| pVAJ2-2 | pVA380::(*rnjB* [NTD]-3x FLAG), Em^r^ | This study |
| pVAJ2-3 | pVA380::(*gfp* [Superfolder]-*rnjB* [CTD]-3x FLAG), Em^r^ | Unpublished |
| pET29b-J1J2 | pET29b :: rnja-rnjb-6×His, Km^r^ | Unpublished |
| pJ2F | pET29b::*rnjB-*3x FLAG, Km^r^ | This study |
| pJ1HJ2F | pET29b :: *rnja*-HA_*rnjb*-3×FLAG, Km^r^ | Unpublished |
| pDnaJHJ2F | pET29b::(HA-*dnaJ*, *rnjB-*3x FLAG), Km^r^ | This study |
| pFtsZHJ2F | pET29b::(*ftsZ*-HA, *rnjB-*3x FLAG), Km^r^ | This study |
| pRnYHJ2F | pET29b::(*rny*-HA, *rnjB-*3x FLAG), Km^r^ | This study |
| pDnaJHJ2T | pET29b::(HA-*dnaJ*, *gfp* [NeonGreen]*-rnjB* [CTD]-3x FLAG), Km^r^ | This study |
| pFtsZHJ2T | pET29b::(*ftsZ*-HA, *gfp* [NeonGreen]*-rnjB* [CTD]-3x FLAG), Km^r^ | This study |
| pRnYHJ2T | pET29b::(*rny*-HA, *gfp-rnjB* [CTD]-3x FLAG), Km^r^ | This study |
| pJ2T | pET29b::(*gfp* [NeonGreen]*-rnjB* [CTD]-3x FLAG), Km^r^ | This study |
| pDnaJHG | pET29b::(HA-*dnaJ*, *gfp* [NeonGreen]-3x FLAG), Km^r^ | This study |
| pFtsZHG | pET29b::(*ftsZ*-HA, *gfp* [NeonGreen]-3x FLAG), Km^r^ | This study |
| pRnYHG | pET29b::(*rny*-HA, *gfp* [NeonGreen]-3x FLAG), Km^r^ | This study |
| pG | pET29b::*gfp* [NeonGreen]-3x FLAG, Km^r^ | This study |

*Em^r^, erythromycin resistance; Km^r^, kanamycin resistance; Spec^r^, spectinomycin resistance

Ajdic, D., McShan, W.M., McLaughlin, R.E., Savic, G., Chang, J., Carson, M.B., Primeaux, C., Tian, R., Kenton, S., Jia, H., Lin, S., Qian, Y., Li, S., Zhu, H., Najar, F., Lai, H., White, J., Roe, B.A., and Ferretti, J.J. (2002) Genome sequence of Streptococcus mutans UA159, a cariogenic dental pathogen. *Proceedings of the National Academy of Sciences of the United States of America* **99**: 14434-14439.

Qi, F., Chen, P., and Caufield, P.W. (2001) The group I strain of Streptococcus mutans, UA140, produces both the lantibiotic mutacin I and a nonlantibiotic bacteriocin, mutacin IV. *Applied and environmental microbiology* **67**: 15-21.

Xie, Z., Qi, F., and Merritt, J. (2013) Development of a tunable wide-range gene induction system useful for the study of streptococcal toxin-antitoxin systems. *Applied and environmental microbiology* **79**: 6375-6384.

Zhang, S., Zou, Z., Kreth, J., and Merritt, J. (2017) Recombineering in Streptococcus mutans Using Direct Repeat-Mediated Cloning-Independent Markerless Mutagenesis (DR-CIMM). *Frontiers in cellular and infection microbiology* **7**: 202.

**Table S2.** **Primers used in this study.**

| **Construct and primer** | **Primer sequence (5′-3′)*** |
| --- | --- |
| *Mutants created by CIMM* | |
| For all mutants except for *pnp* | |
| IFDC3F | CCGAGCAACAATAACACTCATAGCATAG |
| IFDC3R | GAAGCTGTCAGTAGTATACCTAATAATTTATC |
| ***rnjB* 3x FLAG** | |
| J2upF1 | CAATTGGGAAGGACGTATTATCGT |
| J2upR1 | **CTATGCTATGAGTGTTATTGTTGCTCGG**TTATCTAACTTCCATAACTACTGGTA |
| J2dnF1 | **GATAAATTATTAGGTATACTACTGACAGCTTC**AAGAGGCTTGATTGATACTGT |
| J2dnR1 | GAGATGCTCCTGTCGTTTCTCCT |
| J2upR2 | **CCTTGTAGTCACCGTCGTGGTCCTTGTAGTC**TCTAACTTCCATAACTACTGGT |
| J2upR3 | **TTACTTGTCGTCGTCGTCCTTGTAGTCGATGTCGTGGT**CCTTGTAGTCACCGTCGTG |
| J2dnF2 | **TACAAGGACGACGACGACAAGTAA**AAGAGGCTTGATTGATACTGTTTC |
| ***rnjA*-HA** | |
| J1upF1 | CATCGAAGGTATTCATGGACGTAT |
| J1upR1 | **CTATGCTATGAGTGTTATTGTTGCTCGG**TTAAGACTTATCTGGTGTCAATATCAT |
| J1dnF1 | **GATAAATTATTAGGTATACTACTGACAGCTTC**ATCATAATTGTAAGCAGCTATTATTAA |
| J1dnR1 | GGAATTCGAGAAGGACGCAAAAC |
| J1upR2 | **TTAAGCGTAATCTGGAACATCGTATGGGTA**AGACTTATCTGGTGTCAATATCATAGG |
| J1dnF2 | **TACCCATACGATGTTCCAGATTACGCTTAA**ATCATAATTGTAAGCAGCTATTATTAA |
| ***cshA*-HA** | |
| CshupF1 | TTGAAGCCATTATTTCACGTGTACC |
| CshupR1 | **TGCTATGAGTGTTATTGTTGCTCGG**TTATCTGTCACCTTTATTGCGAATG |
| CshdnF1 | **GATAAATTATTAGGTATACTACTGACAGCTTC**CCATTCAAATTCTCTTAAAACATATGTT |
| CshdnR1 | CAGTAGCACATGTAAGTAGATAAATACC |
| CshupR2 | **TTAAGCGTAATCTGGAACATCGTATGGGTA**TCTGTCACCTTTATTGCGAATGGT |
| CshdnF2 | **TACCCATACGATGTTCCAGATTACGCTTAA**CCATTCAAATTCTCTTAAAACATATGTTTC |
| ***pnp*-HA** | |
| PnpupF1 | TTCTTGCCAAAAGTACATGGTTCA |
| PnpupR1 | **TGCTATGAGTGTTATTGTTGCTCGG**TTATTTATCATATTTTTCTTTACCTTTATCGTCTT |
| PnpdnF1 | **ACTACTGACAGCTTCAGGAGAAACTT**ATGATAAATAAATTTAATAAAGAACTTGATATGG |
| PnpdnR1 | ACCAATAACTTGTGAATGTGCAGAA |
| IFDC3Rrbs | **AAGTTTCTCCT**GAAGCTGTCAGTAGTATACCTAATAATTTATC |
| PnpupR2 | **TTAAGCGTAATCTGGAACATCGTATGGGTA**TTTATCATATTTTTCTTTACCTTTATCGTC |
| PnpdnF2 | **CATACGATGTTCCAGATTACGCTTAA**AGGAGAAACTTATGATAAATAAATTTAATAAAGA |
| ***rpoB*-HA** | |
| RpoupF1 | AGGTTAAAGAAGGTGATATTCTAGTTG |
| RpoupR1 | **CTATGCTATGAGTGTTATTGTTGCTCGG**TTATTTCTCATCACTATTTGCAAATTCAG |
| RpodnF1 | **TTATTAGGTATACTACTGACAGCTTC**GTGATTTAGTAACTTAGTTTATCTTTTATGATTG |
| RpodnR1 | ACGATTGTTCCGATTGATCACA |
| RpoupR2 | **TTAAGCGTAATCTGGAACATCGTATGGGTA**TTTCTCATCACTATTTGCAAATTCAG |
| RpodnF2 | **TACCCATACGATGTTCCAGATTACGCTTAA**GTGATTTAGTAACTTAGTTTATCTTTTATG |
| ***rny*-HA** | |
| RnyupF1 | TAGCGCATGATATTGCGAATCGTA |
| RnyupR1 | **TGCTATGAGTGTTATTGTTGCTCGG**TCATTTAGCATAATCAATCGCACGA |
| RnydnF1 | **GAATGTAGATAAATTATTAGGTATACTACTGACAGCTTC**GAAGAGCAGGGGTAGGAACAA |
| RnydnR1 | CAAGGTATCAATTGATGGTTTTAACATCAT |
| RnyupR2 | **TCAAGCGTAATCTGGAACATCGTATGGGTA**TTTAGCATAATCAATCGCACGAAGT |
| RnydnF2 | **TACCCATACGATGTTCCAGATTACGCTTGA**GAAGAGCAGGGGTAGGAACAA |
| ***infB*-HA** | |
| InfupF1 | TAGAAACTGTTCTTCTGGTAGCAG |
| InfupR1 | **CTATGCTATGAGTGTTATTGTTGCTCGG**TTATCTTTTAATTTCTTCCATAATGTAAGC |
| InfdnF1 | **ATTATTAGGTATACTACTGACAGCTTC**TAATATTTAGTTACTCCTTTGTTAACACTTC |
| InfdnR1 | AGCCAACCGACATCTCTAATATAG |
| InfupR2 | **TTAAGCGTAATCTGGAACATCGTATGGGTA**TCTTTTAATTTCTTCCATAATGTAAGCCT |
| InfdnF2 | **ACCCATACGATGTTCCAGATTACGCTTAA**TAATATTTAGTTACTCCTTTGTTAACACTTC |
| ***dnaK*-HA** | |
| DKupF1 | CATTTAGAAACGAGCCTTTCTCGT |
| DKupR1 | **CTATGCTATGAGTGTTATTGTTGCTCGG**TTATTTCTCCGTAAACTCTCCATCTACA |
| DKdnF1 | **GATAAATTATTAGGTATACTACTGACAGCTTC**AATAGTTCAGTGGACTATTTTATCCCGA |
| DKdnR1 | CATATTGATAACACCTGAACCATGACA |
| DKupR2 | **TTAAGCGTAATCTGGAACATCGTATGGGTA**TTTCTCCGTAAACTCTCCATCTACA |
| DKdnF2 | **TACCCATACGATGTTCCAGATTACGCTTAA**AATAGTTCAGTGGACTATTTTATCCCGA |
| ***ldh*-HA** | |
| LdhupF1 | GATGTTTAGAACATGACTGCAACT |
| LdhupR1 | **CTATGCTATGAGTGTTATTGTTGCTCGG**TTAGTTACGAGCTGCAGCAGCAA |
| LdhdnF1 | **GATAAATTATTAGGTATACTACTGACAGCTTC**ACAATAAAAATCCATAAAAATACCAAGC |
| LdhdnR1 | GCTACAACAGTACGAAACAGAG |
| LdhupR2 | **TTAAGCGTAATCTGGAACATCGTATGGGTA**GTTACGAGCTGCAGCAGCAA |
| LdhdnF2 | **TACCCATACGATGTTCCAGATTACGCTTAA**ACAATAAAAATCCATAAAAATACCAAGC |
| **SMU_965-HA** | |
| 965upF1 | GTCCGATGACGATATTGCTATTGTT |
| 965upR1 | **CTATGCTATGAGTGTTATTGTTGCTCGG**TTATTCCCCTAATACTTTGAAAGTGTTGAGC |
| 965dnF1 | **GATAAATTATTAGGTATACTACTGACAGCTTC**GATGAAAATTACAGTACCTGCAACATCA |
| 965dnR1 | CCAAGTCAATGCTTCTTGATAATTCAT |
| 965upR2 | **TTAAGCGTAATCTGGAACATCGTATGGGTA**TTCCCCTAATACTTTGAAAGTGTTGAGC |
| 965dnF2 | **TACCCATACGATGTTCCAGATTACGCTTAA**GATGAAAATTACAGTACCTGCAACATCAG |
| ***ftsZ*-HA** | |
| FZupF1 | CAACCTGAAATTGGACGTAAAGCT |
| FZupR1 | **CTATGCTATGAGTGTTATTGTTGCTCGG**TTAACGATTCTTAAAGAAAGGAGGTG |
| FZdnF1 | **GATAAATTATTAGGTATACTACTGACAGCTTC**TAATGGATTTACAAGCAAATAAAGAACA |
| FZdnR1 | CAAATCAACAATCTCTTCAGCATC |
| FZupR2 | **TTAAGCGTAATCTGGAACATCGTATGGGTA**ACGATTCTTAAAGAAAGGAGGTGTTTC |
| FZdnF2 | **TACCCATACGATGTTCCAGATTACGCTTAA**TAATGGATTTACAAGCAAATAAAGAACACG |
| *Mutants created by DR-CIMM* | |
| ***pfkA*-HA** |  |
| PfkupF1 | ATGAAACGTATTGCTGTTTTGACCA |
| PfkupR1 | **CTATGCTATGAGTGTTATTGTTGCTCGG**TTAGTTGGACAAATCACGATTCAAAGCA |
| PfkupF2 | **GTAGATAAATTATTAGGTATACTACTGACAGCTTC**TGAATTGCTCAGGGAAGGAAAAG |
| PfkupR2 | **TTAAGCGTAATCTGGAACATCGTATGGGTA**GTTGGACAAATCACGATTCAAAGCA |
| PfkdnF1 | **TACCCATACGATGTTCCAGATTACGCTTAA**GCATGATTAAGGTCGGTTAGATTATAAGTT |
| PfkdnR1 | TCAGTCATTGTTTCAAGCATATTTGTTG |
| ***eno*-HA** | |
| EnoupF1 | GCTATTGATAAAGCAATGATCGCACT |
| EnoupR1 | **CTATGCTATGAGTGTTATTGTTGCTCGG**TTATTTTTTCAAGTTGTAGAATGATTTAAGACC |
| EnoupF2 | **GTAGATAAATTATTAGGTATACTACTGACAGCTTC**CGTTCAGGTGAAACTGAAGATTCAA |
| EnoupR2 | **TTAAGCGTAATCTGGAACATCGTATGGGTA**TTTTTTCAAGTTGTAGAATGATTTAAGACC |
| EnodnF1 | **TACCCATACGATGTTCCAGATTACGCTTAA**TAAGTTATTTAAAACAAAGAGCGCATCTAA |
| EnodnR1 | AACCAATCCTATCTGAGATAAAGACTTGT |
| *Mutants created by allelic exchange* | |
| **HA-*dnaJ*** |  |
| ErmF1 | GAAGGAGTGATTACATGAACAAAAATA |
| ErmRDJ | **GGTACATATAAGATATTAAGCCGTTAAGCGA**TTATTTCCTCCCGTTAAATAATAGATAAC |
| DJupF1 | GATAAGAACGGTATCGTGTCTGTTAAGG |
| DJupR1 | **TATTTTTGTTCATGTAATCACTCCTTC**CTCAAAGAAAAATAAGAAATCCAACGAAGATAC |
| DJdnF1 | **TACCCATACGATGTTCCAGATTACGCT**AACAATCAAGAATATTATGATCGTCTTGGT |
| DJdnR1 | TAACAGTAACGTGCTGATCTCCCT |
| DJdnF2 | **GGAAATAATCGCTTAACGGCTTAATATCTTATATG**TACCCATACGATGTTCCAGATTACG |
| **HA-*ftsA*** | |
| ErmF1 | GAAGGAGTGATTACATGAACAAAAATA |
| ErmRFA | **CGTATGGGTACATTTACTCGACCTCTCTATC**TTATTTCCTCCCGTTAAATAATAGATAAC |
| FAupF1 | TCAAACTGATAAAGAAGCAGACAGTAAG |
| FAupR1 | **TATTTTTGTTCATGTAATCACTCCTTC**TTTCCAATCTATTACTATTAAAAACTGTCAAAA |
| FAdnF1 | **TACCCATACGATGTTCCAGATTACGCT**GCTAGAGATGGCTTTTTTACAGGATTG |
| FAdnR1 | CGACTTGGTTTGGAACATGAAGTTTAAC |
| FAdnF2 | **AGGAAATAAGATAGAGAGGTCGAGTAAATG**TACCCATACGATGTTCCAGATTACG |
|  | |
| **Superfolder GFP fusion proteins** | |
| sf1F | GACTACAAGGACCACGACGGTGA |
| sf2R | **GTGAACAGCTCTTCTCCTTTTGACAT**GTTCTAAACATCTCCTTTTATTTCCTCCCGTTAA |
| sf3F | ATGTCAAAAGGAGAAGAGCTGTTCACAGGTGTTGTGCC |
| sf4R | **AGTCACCGTCGTGGTCCTTGTAGTC**CGAGCCGCCGCCGCCCTTATAAA |
| **NeonGreen GFP fusion proteins** | |
| ng1F | TCCAACGTGATGAAATCGAAC |
| ng2R | **GTCGTGGTCCTTGTAGTCCGAGCCGCCGCCGCC**TTTATAAAGTTCATCCATACCCATAAC |
| ng3F | GGCTCGGACTACAAGGACCA |
| ng4R | **AATATTTTATATTTTTGTTCATGTTATCACTCCTGAAGTTA**CTTGTCGTCGTCGTCCTTG |
| ng5F | CTTCAGGAGTGATAACATGAACAAAAATATAAAATATTC |
| ng6R | TTCCAGTTACCAGCAATAATTGG |
| ng7R | **CGAGCCGCCGCCGCC**TTTATAAAGTTCATCCATACCCATAACATC |
| ng8F | **ATGGATGAACTTTATAAAGGCGGCGGCGGCTCG**GGTATCTTTATCATTGCCTTAACTGTC |
| ng9F | **TATGGATGAACTTTATAAAGGCGGCGGCGGCTCG**AGTGACATTAAAATTATTGCCCTAGG |
| **Expression vectors** |  |
| su1F | **TACAAGGACGACGACGACAAGTAA**CAGATACGGTAAACTAGCCTCGT |
| su2R | **AAGATACCCATGTTCTAAACATCTCCTT**TTATTTCCTCCCGTTAAATAATAGATAACTAT |
| su3F | **AAGGAGATGTTTAGAACATG**GGTATCTTTATCATTGCCTTAACTGTC |
| su4R | TTACTTGTCGTCGTCGTCCTTGTA |
| su5F | GACTACAAGGACCACGACGGT |
| su6R | CATGTTCTAAACATCTCCTTTTATTTCCTCC |
| su7F | **AAGGAGATGTTTAGAAC**ATGAGTGACATTAAAATTATTGCCCTAG |
| su8R | **ACCGTCGTGGTCCTTGTAGTC**ATCTTCAGAAAGCACCTTGCG |
| p1F | **TCGACTACAAGGACGACGACGACAAG**TGAGATCCGGCTGCTAACAAAGC |
| p2R | CTCCTTCTTAAAGTTAAACAAAATTATTTCTAGAGG |
| p3F | **CTAGAAATAATTTTGTTTAACTTTAAGAAGGAG**ACATAATGAGTGACATTAAAATTATTG |
| p4R | CTTGTCGTCGTCGTCCTTGTAGTC |
| p5F | **CCATACGATGTTCCAGATTACGCTTAAAAGGAG**ACATAATGAGTGACATTAAAATTATTG |
| p6R | **TAAGCGTAATCTGGAACATCGTATGGGTA**AGACTTATCTGGTGTCAATATCATAGGAATG |
| p7F | AAGGAGACATAATGAGTGACATTAAAATTATTGC |
| p8R | ATGTATATCTCCTTCTTAAAGTTAAACAAAATTATTTCTAGAGG |
| p10R | **TAATGTCACTCATTATGTCTCCTT**TTAAGCGTAATCTGGAACATCGTATGGGTA |
| p11F | **TTTGTTTAACTTTAAGAAGGAGATATACAT**ATGTACCCATACGATGTTCCAGATTACGCT |
| p12R | **GCAATAATTTTAATGTCACTCATTATGTCTCCTT**TTACATCTCATCAAAAGCGTCCTTGA |
| p15F | **AAATAATTTTGTTTAACTTTAAGAAGGAGATATACAT**ATGGCATTTTCATTTGATGCAGC |
| p17F | **GTTTAACTTTAAGAAGGAGATATACAT**ATGTTAAATATTCTTTTAACTCTTGTTTTCTCC |
| p18R | **TAATGTCACTCATTATGTCTCCTT**TCAAGCGTAATCTGGAACATCGTATGGGTA |
| p19F | **TGTTCCAGATTACGCTTAAAAGGAGACATA**ATGGTTTCAAAAGGTGAAGAAGATAATATG |
| p20R | CTTGTCGTCGTCGTCCTTGTAGTCGA |
| p21F | **CGCTTTTGATGAGATGTAAAAGGAGACATA**ATGGTTTCAAAAGGTGAAGAAGATAATATG |
| p22F | **TGTTCCAGATTACGCTTGAAAGGAGACATA**ATGGTTTCAAAAGGTGAAGAAGATAATATG |
| p23R | TATGTCTCCTTTTAAGCGTAATCTGGAACA |
| p24R | TATGTCTCCTTTTACATCTCATCAAAAGCG |
| p25R | TATGTCTCCTTTCAAGCGTAATCTGGAACA |
| p26F | **AATTTTGTTTAACTTTAAGAAGGAGACATA**ATGGTTTCAAAAGGTGAAGAAGATAATATG |
| p27R | CTTGTCGTCGTCGTCCTTGTAGTCGA |
| p28R | TATGTCTCCTTCTTAAAGTTAAACAAAATT |
| pZX9F | TAAAAGCTTGATTTTCGTTCGTGAAG |
| pZX9R | CATATTTACCTCCTTTGATTTAAGTGAAC |
| ftsHF1 | **GATTACAAGGACGATGACGATAAG**AAAAATAATCGAAATAATGGATTTGTT |
| ftsHR2 | **CTTCACGAACGAAAATCAAGCTT**TTAATCAGTATTATCTTCAGACATTTTTTC |
| ftsHF3 | **GTTCACTTAAATCAAAGGAGGTAAATATG**GATTACAAGGACGATGACGATAAG |

*Complementary sequences used for overlap extension PCR are shown in bold.

**Fig. S1. Multiple sequence alignment of RNase J family enzymes.**

**O31760:** VRIIALGGVGEIGKNLYVIE[2]SDIFVVDAGLMHPENEMLGIDVVIPDISYLIERADRVKAIFLTHGHDENIGGVFYLLNK-LS---VPV-YGTKLTLALLRE-K--LKQYGHNR[6]IHS---KSVITF-ES :132
**Q49X63:** IRIIPLGGVGEIAKNMYIVE[2]DEMFMLDAGLMFPEDEMLGVDIVIPDIQYVIENKEKLKGIFLTHGHEHAIGAVTYVLEQ-VD---APV-YGSKLTIALIKEN---MKARNVNK[4]VNN---ESVMRF-KG :132
**Q4L5X8:** IRIIPLGGVGEIAKNMYIVE[2]DEMFMLDAGLMFPEDEMLGVDIVIPDIQYVIENKEKLKGIFLSHGHEHAIGAVSYILEQ-ID---APV-YGSKLTLALVKEN---MKSRNVKK[4]VNH---DSVMRF-KN :132
**Q5HPR6:** IRIIPLGGVGEIAKNMYIVE[2]DEMFMLDAGLMFPEDEMLGVDIVIPDIQYVIENKERLKGIFLTHGHEHAIGAVSYVLEQ-ID---APV-YGSKLTIALVKEA---MKARNIKK[4]VNH---DSIMRF-KN :132
**Q6G9T8:** IRIIPLGGVGEIAKNMYIVE[2]DEMFMLDAGLMFPEDEMLGIDIVIPDISYVLENKDKLKGIFLTHGHEHAIGAVSYVLEQ-LD---APV-YGSKLTIALIKEN---MKARNIDK[4]VNN---DSIMRF-KN :132
**Q6GHG0:** IRIIPLGGVGEIAKNMYIVE[2]DEMFMLDAGLMFPEDEMLGIDIVIPDISYVLENKEKLKGIFLTHGHEHAIGAVSYVLEQ-LD---APV-YGSKLTIALIKEN---MKARNIDK[4]VNN---DSIMRF-KN :132
**Q8K7S6:** IKMIALGGVREYGKNFYLVE[2]DSMFILDAGLKYPENEQLGVDLVIPNLDYVIENKGKVQGIFLSHGHADAIGALPYLLAE-VS---APV-FGSELTIELAKL-F--VKSNNSTK[7]VDS---DTEIEF-KD :128

**Q8DTB3:** IKIIALGGVRENGKNFYLVE[2]DAIFILDAGLKYPENEQLGVDVVIPNVDYVIENKKRVQGIFLTHGHADAIGALPYLLSD-VK---APV-FGSELTIELAKLV---VKNNDATK[6]VDA---QTEIEF-DD :127

**Q2YX35:** VGVYALGGLGEIGKNTYAVE[2]DEIVIIDAGIKFPDDNLLGIDYVIPDYTYLVQNQDKIVGLFITHGHEDHIGGVPFLLKQ-LN---IPI-YGGPLALGLIR-NK--LEEHHLLR[6]INE---DSVIKS-KH :132
**Q6GAC5:** VGVYALGGLGEIGKNTYAVE[2]DEIVIIDAGIKFPDDNLLGIDYVIPDYTYLVQNQDKIVGLFITHGHEDHIGGVPFLLKQ-LN---IPI-YGGPLALGLIR-NK--LEEHHLLR[6]INE---DSVIKS-KH :132
**Q4L5A3:** VGVYALGGLGEVGKNTYAIE[2]NEIVIIDAGIKFPDDNLLGIDYVIPDYTYLEQNQDKIVGLFITHGHEDHIGGVPYLLKQ-IN---VPI-YGGPLALGLIR-NK--LDEHNLLR[6]ITE---DSVIKS-KH :132
**Q5HQ80:** VGVYALGGLGEVGKNTYAVE[2]DEIVIIDAGIKFPDDNLLGIDYVIPDYTYLEQNQDKIVGLFITHGHEDHIGGVPFLLKQ-IN---VPI-YGGPLALGLIR-NK--LEEHHLLR[6]IDE---SSVIKS-KH :132
**Q49WL3:** VAVYALGGLGEIGKNTYAVE[2]DEIVIIDAGIKFPDDNLLGIDYVIPDITYLEQNQDKIVGLFITHGHEDHIGGVPYLLKQ-IN---VPI-YGGPLALGLIR-NK--LEEHHLLR[6]IDE---SSVIKS-KH :132
**Q45493:** TAVFALGGLGEIGKNTYAVQ[2]DEIVLIDAGIKFPEDELLGIDYVIPDYTYLVKNEDKIKGLFITHGHEDHIGGIPYLLRQ-VN---IPV-YGGKLAIGLLR-NK--LEEHGLLR[6]IGE---DDIVKF-RK :132
**Q8K5W8:** VGVFAIGGLGEIGKNTYGIE[2]DEIIIVDAGIKFPEDDLLGIDYVIPDYSYIVDNLDRVKALVITHGHEDHIGGIPFLLKQ-AN---IPI-YAGPLALALIRG-K--LEEHGLWR[6]INH---NTELTF-KN :134
**Q8DVU7:** VGVYAIGGLGEIGKNTYGIE[2]DEIIIVDAGIKFPEDDLLGIDYVIPDYSYIVDNVDRVKALVITHGHEDHIGGIPFLLKQ-AS---IPI-YAGPLALALIRG-K--LEEHGLWR[6]INH---NTELTF-KN :134

**P54123:** LKILPLGGLHEIGKNTCVFE[2]DEILLLDAGLAFPTDDMHGVNVVLPDMTYLRENREKIKGMVVTHGHEDHIGGIAYHLKQ-FD---IPIIYGPRLAMALLRD-K--LEEAGMLE[6]VSP---REMVRL-GK :134
**P56185:** VKITPLGGLGEIGGNMMVIE[2]KSAIVIDAGMSFPKEGLFGVDILIPDFSYLHQIKDKIAGIIITHAHEDHIGATPYLFKE-LQ---FPL-YGTPLSLGLIG-SK--FDEHGLKK[7]VEK[3]ISVGEF--- :266
**A0QVT2:** LRVTALGGISEIGRNMTVFE[2]GRLLIVDCGVLFPGHDEPGVDLILPDLRHIEDRLDEIEALVVTHAHEDHIGAIPFLLKLRPD---IPV-VGSKFTIALVRE-K--CREHRLKP[4]VAE---RQSSQH-GV :138
**P9WGZ8:** LRVTALGGINEIGRNMTVFE[2]GRLLIIDCGVLFPGHDEPGVDLILPDMRHVEDRLDDIEALVLTHGHEDHIGAIPFLLKLRPD---IPV-VGSKFTLALVAE-K--CREYRITP[4]VRE---GQSTRH-GV :138
**P54122:** LRIYALGGISEIGRNMTVFE[2]NRLLIVDCGVLFPSSGEPGVDLILPDFGPIEDHLHRVDALVVTHGHEDHIGAIPWLLKLRND---IPI-LASRFTLALIAA-K--CKEHRQRP[4]VNE[1]SNEDR--GP :277
**M4MR97:** LVFLPLGGVGEIGMNLGLYG[6]RQWIMVDCGVTFPGPELPGVDLVLPDIAFLAEQRRNLKAIIITHAHEDHYGALNDLWPG-LN---VPV-YASPFTAGML-EAKRAFEKSRSEI[3]IFK[1]GDRINV-GP :143
**Q72JJ7:** VEIIPLGGMGEIGKNITVFR[2]DEIFVLDGGLAFPEEGMPGVDLLIPRVDYLIEHRHKIKAWVLTHGHEDHIGGLPFLLPM-IF[5]VPI-YGARLTLGLLRG-K--LEEFGLRP[7]ISP---DDRIQVGRY :158

**O31760:** T-KVSFFRTIHSIPDSVGVSFKTSLGSIVCTGDFKFDQTPALNQ--TC-DIGEIAKIGNSGVLALLSDSANA-ERPGYTPSEAAVSG-EISDALYNS-QNRVIIAVFASNINRIQQVIHAAAQNGRKIAVAGK : 258

**Q49X63:** V-NVTFFNTTHSIPDSLGICIHTSYGAIVYTGEFKFDQS-LHGHYAP--DLKKMTEIGEAGVFALISDSTEA-EKPGYNTPENVI-ESHMYDA-FTKVKGRLIVSCYASNFIRIQQVLNLAQRLNRKVSFLGR : 258

**Q4L5X8:** V-NITFFNTTHSIPDSLGICIHTSYGSIVYTGEFKFDQS-LQGHYTP--DIKRMAEIGEEGVFALISDSTEA-EKPGYNTPENVI-ESHIYDA-FAKVKGRLIVSCYASNFIRIQQVLNTASKLNRKVSFLGR : 258

**Q5HPR6:** V-NVSFFNTTHSIPDSLGVCIHTSYGSIVYTGEFKFDQS-LHGHYAP--DLKRMAEIGDEGVFALISDSTEA-EKPGYNTPENII-EHHMYDA-FAKVKGRLIVSCYASNFVRIQQVLNIASQLNRKVSFLGR : 258

**Q6G9T8:** V-NISFFNTTHSIPDSLGVCIHTSYGAIVYTGEFKFDQS-LHGHYAP--DIKRMAEIGEEGVFVLISDSTEA-EKPGYNTPENVI-EHHMYDA-FAKVRGRLIVSCYASNFIRIQQVLNIASKLNRKVSFLGR : 258

**Q6GHG0:** V-NISFFNTTHSIPDSLGVCIHTSYGAIVYTGEFKFDQS-LHGHYAP--DIKRMAEIGEEGVFVLISDSTEA-EKPGYNTPENVI-EHHMYDA-FAKVRGRLIVSCYASNFIRIQQVLNIASKLNRKVSFLGR : 258

**Q8K7S6:** G-LVSFFRTTHSIPESMGIVIGTDKGNIVYTGDFKFDQAAREG-Y-QT-DLLRLAEIGKEGVLALLSDSVNATSNDQIA-SESEV-GEEMDSV-ISDADGRVIVAAVASNLVRIQQVFDSATAHGRRVVLTGT : 254

**Q8DTB3:** A-IISFFKTTHSIPESLGIVVGTKEGNIVYTGDFKFDQAA—SKLY-QT-DLARIAEIGREGVLALLSDSANA-DSTVQIASEAEV-GIEMEQV-ISNWEGRVIVAAVASNLVRIQQVFDSAANNGRRVVLTGY : 253

**Q2YX35:** L-TISFYLTTHSIPETYGVIVDTPEGKVVHTGDFKFDFTPV-GK--PA-NIAKMAQLGEEGVLCLLSDSTNS-LVPDFTLSEREV-GQNVDKI-FRNCKGRIIFATFASNIYRVQQAVEAAIKNNRKIVTFGR : 257

**Q6GAC5:** F-TISFYLTTHSIPETYGVIVDTPEGKVVHTGDFKFDFTPV-GK--PA-NIAKMAQLGEEGVLCLLSDSTNS-LVPDFTLSEREV-GQNVDKI-FRNCKGRIIFATFASNIYRVQQAVEAAIKNNRKIVTFGR : 257

**Q4L5A3:** F-EISFYLTTHSIPEAYGVIIDTPEGKIVHTGDFKFDFTPV-GE--PA-NIAKMAELGKEGVLCLLSDSTNA-LVPDFTLSEREV-GQNVDKI-FRNCKGRIIFATFASNIYRVQQAVEAAIKYNRKIVTFGR : 257

**Q5HQ80:** F-EISFYLTTHSIPEAYGVIVDTPEGKIVHTGDFKFDFTPV-GE--PA-NIAKMAQLGHEGVLCLLSDSTNA-LVPDFTLSEREV-GQNVDKI-FRNCKGRIIFATFASNIYRVQQAVEAAIKYNRKIVTFGR : 257

**Q49WL3:** F-EVSFYLTTHSIPEAYGVIVNTPEGNIVHTGDFKFDFTPV-GE--PA-NIAKMAKLGEEGVLCLLSDSTNS-LVPDFTLSEREV-GQNVEKI-FRNCSGRIIFATFASNIYRVQQAVEAAIKHNRKIVTFGR : 257

**Q45493:** T-AVSFFRTTHSIPDSYGIVVKTPPGNIVHTGDFKFDFTPV-GE--PA-NLTKMAEIGKEGVLCLLSDSTNS-ENPEFTMSERRV-GESIHDI-FRKVDGRIIFATFASNIHRLQQVIEAAVQNGRKVAVFGR : 257

**Q8K5W8:** M-SVTFFKTTHSIPEPVGIVIHTPQGKIICTGDFKFDFTPV-GD--PA-DLQRMAALGEEGVLCLLSDSTNA-EIPTFTNSEKVV-GQSILKI-IEGIHGRIIFASFASNIYRLQQAAEAAVKTGRKIAVFGR : 259

**Q8DVU7:** L-SVTFFRTTHSIPEPLGIVIHTPQGKVVCTGDFKFDWTPV-GE--PA-DIHRMAALGEDGVLCLLSDSTNA-EIPTFTNSEKVV-GQSIMNI-IEGIHGRIIFASFASNIFRLQQAAEAAVKTGRKIAVFGR : 259

**P54123:** SFVVEFIRNTHSIADSYCLAIHTPLGVVMHSGDFKIDHTPIDGE--FF-DLQKVAEYGEKGVLCLLSDSTNA-EVPGITPSEASV-IPNLDRV-FSQAEGRLMVTTFASSVHRVNIILSLAQKHQRKVAVVGR : 261

**P56185:** --IIEWIHITHSIIDSSALAIQTKAGTIIHTGDFKIDHTPV--DNLPT-DLYRLAHYGEKGVMLLLSDSTNS-HKSGTTPSESTI-APAFDTL-FKEAQGRVIMSTFSSNIHRVYQAIQYGIKYNRKIAVIGR : 391

**A0QVT2:** F-ECEYFAVNHSIPGCLAVAIHTGAGTVLHTGDIKLDQLPLDGR--PT-DLPGMSRLGDAGVDLFLCDSTNS-EHPGVSPSESEV-GPTLHRL-IRGAEGRVIVACFASNVDRVQQIIDAAVALGRRVSFVGR : 264

**P9WGZ8:** F-ECEYFAVNHSTPDALAIAVYTGAGTILHTGDIKFDQLPPDGR--PT-DLPGMSRLGDTGVDLLLCDSTNA-EIPGVGPSESEV-GPTLHRL-IRGADGRVIVACFASNVDRVQQIIDAAVALGRRVSFVGR : 264

**P54122:** F-NIRFWAVNHSIPDCLGLAIKTPAGLVIHTGDIKLDQTPPDGR--PT-DLPALSRFGDEGVDLMLCDSTNA-TTPGVSGSEADV-APTLKRL-VGDAKQRVILASFASNVYRVQAAVDAAVASNRKVAFNGR : 403

**M4MR97:** F-SVEAVGVNHSIPEPMALVIRTQLGTVVHTGDWKIDLEPSLG---PLTDESRFRQIGEEGVLALVCDSTNA-LREGVSPSERQV-SESLAKI-IADAEGRVGITTFSSNVGRIRSVAEAAEAAGREVLLLGS : 269

**Q72JJ7:** F-TLDLFRMTHSIPDNSGVVIRTPIGTIVHTGDFKLDPTPIDGK--VS-HLAKVAQAGAEGVLLLIADATNA-ERPGYTPSEMEI-AKELDRV-IGRAPGRVFVTTFASHIHRIQSVIWAAEKYGRKVAMEGR : 284

**O31760:** NLQ[2]LQLARKLGYIE-ADDELFISVQDVKK--YPKREVAIITAGSQGEPLAALTRMANKAHKQLNIEE---GDTVVIASTPIPGQE-LIYSKTVDLLARAGAQVIFAQ--K-R--VHVSGHG-SQEELKL : 376 :
**Q49X63:** SLE[2]FNIARKMGYFD-ISKDLLIPINEVEN--YPKNEVIIIATGMQGEPVEALSQMAQKKHKIMNIEP---GDSV-FLTITASANMEVIVGNTLNELVRAGAEII--P---NSKKIHASSHG-CMEELKM : 376
**Q4L5X8:** SLE[2]FNIARKMGYFD-IPKDLLIPINEVGN--YPKNEVVIIATGMEGEPIEALSQMAQQKHRIMNIEE---GDSV-YLAITASANMEVIIADTLNELVRAGAYII--P--NNK-KIHASSHG-CMEELKM : 376
**Q5HPR6:** SLE[2]FNIARKMGYFD-IPKDLLIPINEVEN--YPKNEVIIIATGMQGEPVEALSQMARKKHKIMNIEE---GDSI-FLAITASANMEVIIADTLNELVRAGAHII--P--NNK-KIHASSHG-CMEELKM : 376 **Q6G9T8:** SLE[2]FNIARKMGYFD-IPKDLLIPITEVDN--YPKNEVIIIATGMQGEPVEALSQMAQHKHKIMNIEE---GDSV-FLAITASANMEVIIANTLNELVRAGAHII--P--NNK-KIHASSHG-CMEELKM : 376 **Q6GHG0:** SLE[2]FNIARKMGYFD-IPKDLLIPITEVDN--YPKNEVIIIATGMQGEPVEALSQMAQHKHKIMNIEE---GDSV-FLAITASANMEVIIANTLNELVRAGAHII--P--NNK-KIHASSHG-CMEELKM : 376
**Q8K7S6:** DAE[6]LRL-EKL-MIT--DERLLIKPKDMSK--FEDHELIILEAGRMGEPINSLQKMAAGRHRYVQIKE---GDLVYIVTTPSTAKE-AMVARVENLIYKAGGSV---KLITQN--LRVSGHANGRDLQ-L : 373
**Q8DTB3:** DVE[5]IRL-KKLSLV---DEKLIVKPKDMHK--FEDHELIILETGRMSEPLNGLRKMAIGRHRYVEIKD---GDLVYIVTTPTVSKE-AVVARVENMIYKAGGDV--RL--TQD--LRVSGHANGRDLQLM : 371

**Q2YX35:** SME[2]IKIGMELGYIK-APPETFIEPNKI-NT-VPKHELLILCTGSQGEPMAALSRIANGTHKQIKIIP---EDTVVFSSSPIPGNT-KSINRTINSLYKAGADVIHSK--ISN--IHTSGHG-SQGDQQL : 376
**Q6GAC5:** SME[2]IKIGMELGYIK-APPETFIEPNKI-NT-VPKHELLILCTGSQGEPMAALSRIANGTHKQIKIIP---EDTVVFSSSPIPGNT-KSINRTINSLYKAGADVIHSK--ISN--IHTSGHG-SQGDQQL : 376
**Q4L5A3:** SME[2]IKIGMELGYIK-APPETFVEPNKI-NN-IPKHELLILCTGSQGEPMAALSRIANGTHKQIKIIP---DDTVVFSSSPIPGNT-KSINRTINALYKAGADVIHSK--ISN--IHTSGHG-SQGDQQL : 376
**Q5HQ80:** SME[2]IKIGMELGYIK-APPETFIEPNKI-NS-VPKHELLILCTGSQGEPMAALSRIANGTHKQIKIIP---EDTVVFSSSPIPGNT-KSINRTINALYKAGADVIHSK--ISN--IHTSGHG-SQGDQQL : 376
**Q49WL3:** SME[2]IKIGMELGYIK-APPETFVEPNKI-NT-VPKHELLILCTGSQGEPMAALSRIANGTHKQIKIIP---EDTVVFSSSPIPGNT-KSINRTINSLYRAGAEVIHNK--VSN--IHTSGHG-SKGDQQL : 376
**Q45493:** SME[2]IEIGQTLGYIN-CPKNTFIEHNEI-NR-MPANKVTILCTGSQGEPMAALSRIANGTHRQISINP---GDTVVFSSSPIPGNT-ISVSRTINQLYRAGAEVIHGP--LND--IHTSGHG-GQEEQKL : 376
**Q8K5W8:** SME[2]IVNGIELGYIK-VPKGTFIEPSELKN--LHASEVLIMCTGSQGESMAALARIANGTHRQVTLQP---GDTVIFSSSPIPGNT-TSVNKLINTIQEAGVDVIHGK--VNN--IHTSGHG-GQQEQKL : 378
**Q8DVU7:** SME[2]IVNGIELGYIK-VPKGTFIEPNEIKE--YHASEIMIMCTGSQGESMAALSRIANGTHRQVTLQP---GDTVIFSSSPIPGNT-TSVNKLINTIQEAGVEVIHGK--VNN--IHTSGHG-GQQEQKL : 378

**P54123:** SML[2]IAHARKLGYIK-CPDNLFVPLKAARN--LPDQQQLILTTGSQGEPLAAMTRISNGEHPQIKIRQ---GDTVVFSANPIPGNT-IAVVNTIDRLMMQGANVIYGK--HQG--IHVSGHA-SQEEHKM : 380
**P56185:** SME[2]LDIARELGYIH-LPYQSFIEANEV-AK-YPDNEILIVTTGSQGETMSALYRMATDEHRHISIKP---NDLVIISAKAIPGNE-ASVSAVLNFLIKKEAKVAYQE--FDN--IHVSGHA-AQEEQKL : 510
**A0QVT2:** SMV[2]MGIARELGYLK-VDDSDILDIAAAEM--MPPDRVVLITTGTQGEPMAALSRMSRGEHRSITLTS---GDLIILSSSLIPGNE-EAVYGVIDSLSKIGARVVTNA--QAR--VHVSGHA-YAGELLF : 383
**P9WGZ8:** SMV[2]MRVARQLGFLR-VADSDLIDIAAAETM-APD-QVVLITTGTQGEPMSALSRMSRGEHRSITLTA---GDLIVLSSSLIPGNE-EAVFGVIDALSKIGARVVTNA--QAR--VHVSGHA-YAGELLF : 383
**P54122:** SMI[2]MEIAEKLGYLK-APRGTII---SMDD--YP-HKVMLITTGTQGEPMAALSRMARREHRQITVRD---GDLIILSSSLVPGNE-EAVFGVINMLAQIGATVV-TG-RDAK--VHTSGHG-YSGELLF : 522
**M4MR97:** SMK[2]VDVARDVGLMEGVKPFLAED--EFGY--IPRDKVVVILTGSQGEPRAALAKIA-RD-E-MRNVAFSAGDTIVFSSRTIPGNE-KAINDIKNGLIEQGIHIITDS--EAL--VHVSGHP-RRTELQQ : 387
**Q72JJ7:** SML[2]SRIALELGYLK-VKD-RLYTLEEVKD--LPDHQVLILATGSQGQPMSVLHRLAFEGHAKMAIKP---GDTVILSSSPIPGNE-EAVNRVINRLYALGAYVLYPP--TYK--VHASGHA-SQEELKL : 402

**O31760:** MINLLKPKYLIPVNGEYRMQKAHSKIAEETGM-[2]SDIF---LIE---KGD----VV----EFR----GQNVKIG---DKVPY----GN---ILIDG-LGVGDIGNIVLRDRRLLSQDGILIVVITLD--- : 474

**Q49X63:** MINIMKPEYFIPVNGEFKMQISHAKLANEAGV-[2]EKIF---LVE---KGD----VV----NYD----GEEMILN---EKVNS----GN---VLIDG-IGVGDVGNIVLRDRHLLAEDGIFIAVVTLD--- : 474

**Q4L5X8:** MINIMKPEYFIPVQGEFKMQIAHAKLANESGV-[2]EKIF---LVE---KGD----VI----HYD----GKDMVLN---EKVNS----GN---ILIDG-IGVGDVGNIVLRDRHLLAEDGIFIAVVTLD--- : 474

**Q5HPR6:** MLNIMKPEYFVPVQGEFKMQIAHAKLAAETGV-[2]EKIF---LVE---KGD----VI----SYN----GKDMILN---EKVQS----GN---ILIDG-IGVGDVGNIVLRDRHLLAEDGIFIAVVTLD--- : 474

**Q6G9T8:** MINIMKPEYFIPVQGEFKMQIAHAKLAAEAGV-[2]EKIF---LVE---KGD----VI----NYN----GKDMILN---EKVNS----GN---ILIDG-IGIGDVGNIVLRDRHLLAEDGIFIAVVTLD--- : 474

**Q6GHG0:** MINIMKPEYFIPVQGEFKMQIAHAKLAAEAGV-[2]EKIF---LVE---KGD----VI----NYN----GKDMILN---EKVNS----GN---ILIDG-IGIGDVGNIVLRDRHLLAEDGIFIAVVTLD--- : 474

**Q8K7S6:** LMNLLKPQYLFPVQGEYRDLAAHAKLAEEVGI----ENIH---ILK---RGD----IM----VLN-----DEGFLH[1]GGVPA----SD---VMIDG-NAIGDVGNIVLRDRKVLSEDGIFIVAITVS--- : 469

**Q8DTB3:** IN-LLKPKYLFPIQGEYRELAAHADLALEIGM-[2]ENIY---IMK---HGD----IM----VYD----KEDGFLHE--GGIPA----GD---VMIDG-NAIGDVGNIVLRDRKVLSEDGIFIIALTVN--- : 469

**Q2YX35:** MLRLIKPKYFLPIHGEYRMLKAHGETGVECGV-[2]DNVF---IFD---IGD----VL----ALT----HDSARKA---GRIPS----GN---VLVDG-SGIGDIGNVVIRDRKLLSEEGLVIVVVSID--- : 474

**Q6GAC5:** MLRLIKPKYFLPIHGEYRMLKAHGETGVECGV-[2]DNVF---IFD---IGD----VL----ALT----HDSARKA---GRIPS----GN---VLVDG-SGIGDIGNVVIRDRKLLSEEGLVIVVVSID--- : 474

**Q4L5A3:** MLRLIQPKYFLPIHGEYRMLKAHGQTGVDCGV-[2]ENVF---IFD---IGD----VL----ALT----HDSARKA---GRIPS----GN---VLVDG-SGIGDIGNVVIRDRKLLSEEGLVIVVVSID--- : 474

**Q5HQ80:** MLRLIQPKYFLPIHGEYRMLKAHGETGVQCGV-[2]DNVF---IFD---IGD----VL----ALT----HDSARKA---GRIPS----GN---VLVDG-SGIGDIGNVVIRDRKLLSEEGLVIVVVSID--- : 474

**Q49WL3:** MLRLLRPKFFLPIHGEYRMLKAHGQSGIECGV-[2]DNVF---IFD---IGD----VL----ALT----HDSARKA---GRIPS----GN---VLVDG-SGIGDIGNVVIRDRKLLSEEGLVIVVVSID--- : 474

**Q45493:** MLRLIKPKFFMPIHGEYRMQKMHVKLATDCGI-[2]ENCF---IMD---NGE----VL----ALK----GDEASVA---GKIPS----GS---VYIDG-SGIGDIGNIVLRDRRILSEEGLVIVVVSID--- : 474

**Q8K5W8:** MLSLIKPKYFMPVHGEYRMQKIHAGLAMDIGI-[2]ENIF---IME---NGD----VL----ALT----SDSARIA---GHFNA----QD---IYVDG-NGIGDIGAAVLRDRRDLSEDGVVLAVATVD--- : 476

**Q8DVU7:** MLRLIKPKYFMPVHGEYRMQKVHAGLAMDIGI-[2]DNIF---IME---NGD----VL----ALT----KDSARRA---GHFNA----QD---IYVDG-NGIGDIGTAVLRDRHDLSEDGVVLAVATVD--- : 476

**P54123:** LLALTRPKFFVPVHGEHRMLVKHSQMAQAQGI-[2]ENIV---IVN---NGD----VI----ELT----GDRIRVA---GQVPS----GI---ELVDQ-AGI--VHESTMAERQQMAEDGLVTVAAALS--- : 476

**P56185:** MLRLIKPKFFLPVHGEYNHVARHKQTAISCGV-[2]KNIY---LME---DGD----QV----EV-----GPAFIKK[1]GTIKS----GK---SYVDNQSNLS-IDTSIVQQREEVASAGVFVATIFVN[6] : 614

**A0QVT2:** LYNGVRPRNVMPVHGTWRHLRANAALAASTGV-[2]ENIV---LAE---NGV----SV----DLV----AGRASIS---GAVTV----GK---MFVDG-LITGDVGDATLGERLI----------------- : 467

**P9WGZ8:** LYNGVRPRNVMPVHGTWRMLRANAKLAASTGV-[2]ESIL---LAE---NGV----SV----DLV----AGKASIS---GAVPV----GK---MFVDG-LIAGDVGDITLGERLI----------------- : 467

**P54122:** LYNAARPKNAMPVHGEWRHLRANKELAISTGV-[2]DNVV---LAQ---NGV----VV----DMV----NGRAQVV---GQIPV----GN---LYVDG-VTMGDIDADILADRTS----------------- : 606

**M4MR97:** MYQWVKPQILVPVHGEAAHLTAHAELGLQSGI[27]GRIY[5]IGDFEEMGI[15]VV[11]DVV[10]EGEAMED[5]GAVES[28]GK[2]VFITK-V-------------------------------- : 564

**Q72JJ7:** ILNLTTPRFFLPWHGEVRHQMNFKWLAESMSR-[2]EKTL---IGE---NGA----VY----RLT----RETFEKV---GEVPH----GV---LYVDG-LGVGDITEEILADRRHMAEEGLVVITALAG--- : 500

**O31760:** KQKKHLVSGPEIITRGFVYVRESEGLIVQA-TELVRS---IVTEAT-E---TSNVE--W[2]-LK[2]M----RDA----LNQF--LYEKT---KRKP--MII---PI-IMEV : 555
**Q49X63:** PKNRRIAAGPEIQSRGFVYVRESEALLNEA-EEKVRE---IVELGL-Q---EKRIE--W[2]-IK[2]M----RDQ----ISKL--LFENT---KRRP--MII---PV-ISEI : 557
**Q4L5X8:** PKNRRIAAGPEIQSRGFV-------YVRES-EELMKEAEDKVR---EIVE[5]RIE--W[2]-IK[2]M----RDQ----ISKL--LFEST---KRRP--MII---PV-ISEI : 557
**Q5HPR6:** PKNRRIAAGPEIQSRGFV-------YVRES-EELLKEAEEKVRKIVEEGLQEKRIE--W[2]-IK[2]M----RDQ----ISKL--LFEST---KRRP--MII---PV-ISEI : 557
**Q6G9T8:** PKNRRIAAGPEIQSRGFVYVRESEDLLREA-EEKVRE---IVEAGL-Q-E--KRIE--W[2]-IK[2]M----RDQ----ISKL--LFEST---KRRP--MII---PV-ISEI : 557
**Q6GHG0:** PKNRRIAAGPEIQSRGFVYVRESEDLLREA-EEKVRE---IVEAGL-Q-E--KRIE--W[2]-IK[2]M----RDQ----ISKL--LFEST---KRRP--MII---PV-ISEI : 557
**Q8K7S6:** KKEKRIISKAKVNTRGFVYVKKSHDILRES-AELVNTTVNYLKK--------DTFD--W[1]-LK[2]V----RDD----LSKF--LFEQT---KRRP--AIL---PV-VMEV : 552
**Q8DTB3:** KKEKKIISRAKINTRGFVYVRKSRDILRES-ADIVNKA---VENYL-Q---QDTFD--W-GE-LKS--I----VRDE---IGKF--LFEQT---KRRP--AIL---PV-VM-- : 551

**Q2YX35:** FNTNKLLSGPDIISRGFVYMRESGQLIYDA-QRKIKTD--VISKLN-QN---KDIQ--W[2]-IK[2]I----IET----LQP---LFEKT---ARKP--MIL---PV-IMKV : 556
**Q6GAC5:** FNTNKLLSGPDIISRGFVYMRESGQLIYDA-QRKIKTD--VISKLN-QN---KDIQ--W[2]-IK[2]I----IET----LQP---LFEKT---ARKP--MIL---PV-IMKV : 556
**Q4L5A3:** FNTNKLLSGPDIISRGFVYMRESGQLIYDA-QRKIKTD--VISKLN-AN---PNIQ--W[2]-IK[2]I----IET----LQP---LYDKT---ARRP--MIL---PV-IMKV : 556
**Q5HQ80:** FNTNKLLSGPDIISRGFVYMRESGQLIYDA-QRKIKGD--VISKLN-SN---KDIQ--W[2]-IK[2]I----IET----LHP---LYEKT---ARKP--MIL---PV-IMKV : 556
**Q49WL3:** FKTNKLLSGPDIISRGFVYMRESGQLIYDA-QRRIKTD--VIGKLN-QN---QDIQ--W[2]-IK[2]I----IET----LQP---LFEKT---ARKP--MIL---PV-IMKV : 556
**Q45493:** MDDFKISAGPDLISRGFVYMRESGDLINDA-QELISNH---LQKVM-ER---KTTQ--W[2]-IK[2]I----TDT----LAP---LYEKT---KRRP--MIL---PI-IMEV : 555
**Q8K5W8:** FNTQMILAGPDILSRGFIYMRESGDLIRES-QRVLFNA---IRIAL-KN---KDAS--I[2]-VN[2]I----VNA----LRP---LYEKT---EREP--III---PM-VLTP : 557
**Q8DVU7:** FKTKMILAGPDILSRGFIYMRESGDLIRSS-QRILFNA---IRIAL-KN---KEAS--I[2]-VN[2]I----VNA----LRPF--LYEKT---EREP--III---PM-IL-- : 555

**P54123:** KTG-TLLAYPEVHCRGVV-MTIQPKLL----EELIVRT---IENFLTER[2]GSTEVSW[14]IK[2]L[11]TDT[16]PAP[2]VLTKT[5]KAKPEKKVV[8]PVSTTKV : 626
**P56185:** LESSQFSS-LGLV--GF---KDEKPLI----KE-IQGG---LEVLL------KSS------AEIL[5]L----EDH----TRN[5]LFKKF---RKYP--AII-------CHA : 686
**A0QVT2:** ----------------------------------------------------------------------------------------------------------------- : 467
**P9WGZ8:** ----------------------------------------------------------------------------------------------------------------- : 467
**P54122:** ----------------------------------------------------------------------------------------------------------------- : 606
**M4MR97:** ----------------------------------------------------------------------------------------------------------------- : 564
**Q72JJ7:** --EDPVV---EVVSRGFVKA--GERLLGEVRRMALEA----LKNGVREK---KPLE--R----IR[4]Y----PVK----KF----LKKAT---GRDP--MIL---PV----- : 569

**Fig. S1. Multiple sequence alignment of RNase J enzymes.** The multiple sequence alignment employed for ConSurf scoring is presented. Homologous sequences were obtained from UniProt, screened for redundancy, aligned using T-Coffee, and manually curated. Large insertions and deletions are denoted in red. Strictly conserved residues are shown in black, and highly conserved residues are shown in grey. Proteins are listed by their respective UniProt accession numbers and are color coded as follows: red (RNase J2), blue (RNase J1), and green (RNase J).
